# Integration of inherent and induced chirality into subphthalocyanine analogue

**DOI:** 10.1038/srep28026

**Published:** 2016-06-13

**Authors:** Luyang Zhao, Dongdong Qi, Kang Wang, Tianyu Wang, Bing Han, Zhiyong Tang, Jianzhuang Jiang

**Affiliations:** 1 Beijing Key Laboratory for Science and Application of Functional Molecular and Crystalline Materials, Department of Chemistry, University of Science and Technology Beijing, Beijing 100083, China; 2 Laboratory of Nanomaterials, National Center for Nanoscience and Technology, Beijing 100190, China

## Abstract

Conventional conjugated systems are characteristic of only either inherent or induced chirality because of synthetic challenge in combination of chiral segment into the main chromophore. In this work, chiral binaphthyl segment is directly fused into the central chromophore of a subphthalocyanine skeleton, resulting in a novel type of chiral subphthalocyanine analogue (*R*/*S*)-**1** of integrated inherent and induced chirality. Impressively, an obviously enhanced optical activity is discerned for (*R*/*S*)-**1** molecules, and corresponding enhancement mechanism is elucidated in detail. The synthesis strategy based on rational molecular design will open the door towards fabrication of chiral materials with giant optical activity, which will have great potential in chiroptical devices.

Chirality describes the geometric property of an object being non-superposable on its mirror image, which is omnipresent in nature^1^,^2^. Due to the major role of the naturally occurring chlorophyll and vitamin B_12_ in life science, chiral porphyrins and their artificial analogues, chiral phthalocyanines, have attracted extensive research interest in the past several decades^3^,^4^,^5^. Study in this area has been significantly focused on exploration of varied molecular structures with strong optical activity in the UV-Vis region, owing to not only unlocking the mystery of life but also their potential application in chiroptical devices^6^. Similarly to other chiral conjugated systems with planar molecular structure^7^, the chirality of porphyrins or phthalocyanines is generally produced *via* two ways: (1) chiral arrangement of the achiral pyrrole/isoindole segments with the help of substituents, endowing the whole molecule with the inherent chirality^8^,^9^; or (2) covalent attachment of additional chiral conjugated fragments onto the tetrapyrrole periphery, resulting in so-called induced chirality^10^,^11^. Nevertheless, the lack of chiral segment in the central tetrapyrrole chromophore constitutes the primary and common feature of all the reported chiral porphyrins and phthalocyanines, regardless of their either inherent or induced chirality nature. As a result, only the relatively weak optical activity is discerned on these chiral tetrapyrrole derivatives, which in turn severely limits their potential applications in chiroptical field^2^,^12^. Thus, it is highly desirable for creation of chiral conjugated systems with intrinsically structural difference from conventional analogues, development of theoretical models to guide design of chiral molecules with strong optical response, and achievement of giant optical activity from new chiral structures.

The ring-contracted homologue of phthalocyanine, subphthalocyanine, is known to possess a bowl-shaped geometrical structure, so it is expected to easily produce chirality compared with phthalocyanine due to absence of a symmetrical plane^13^,^14^,^15^,^16^,^17^,^18^. As clearly demonstrated in Fig. 1(a), chiral arrangement of the central triisoindole/tripyrrole with the help of peripheral substituents (symbol “X” in (a)-1, (a)-2 and (a)-3) makes these molecules generate inherent chiral asymmetry, *C*_3_. Alternatively, incorporation of three chiral binaphthyl substituents onto the subphthalocyanine periphery affords the first but the sole example of subphthalocyanine with induced chirality (Fig. 1(b))^15^. Similarly to the ring-expanded tetrapyrrole homologues, all these subphthalocyanines with either inherent or induced chirality nature exhibit weak molecular dissymmetry as evaluated by the small value of their anisotropic factor *g*, 0.2 × 10^−3^~0.6 × 10^−3^ (Table 1)^15^,^18^,^19^.

Herein, with subphthalocyanine chromophore as the basic conjugated building block, a new strategy is employed towards constructing chiral conjugated system. Direct fusion of the chiral segment, binaphthyl moiety, into the central chromophore of subphthalocyanine skeleton results in a pair of enantiopure isomers (*R*/*S*)-**1** with obviously enhanced optical activity (*g* values reach 0.88 × 10^−3^~3.7 × 10^−3^). It deserves stressing that significantly different from optical response of the conventional chiral subphthalocyanine derivatives, (*R*/*S*)-**1** displays both the inherent chirality characteristics in the Soret band region and the induced chirality in the Q-band range. This work represents the first chiral conjugated system with integrated inherent and induced chirality, which will open a new avenue towards fabrication of novel chiral conjugated systems with enhanced optical activity as well as shed light on possible application in the chiroptical devices.

## Results and Discussion

### Molecular design, synthesis, and characterization

**Figure 1:**
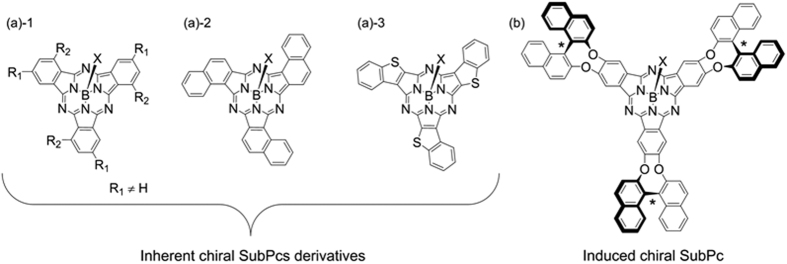
Molecular structures of conventional chiral subphthalocyanine derivatives with inherent (**a**) and induced chirality (**b**). Asterisk indicates locally chiral structure.

**Figure 2:**
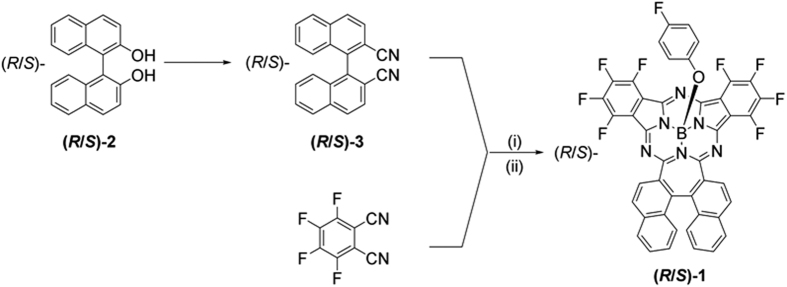
Synthesis of chiral subphthalocyanine analogue (*R*/*S*)-**1**. (i) BBr_3_, chlorobenzene, 60 °C; (ii) 4-Fluorophenol, 90 °C.

Synthesis of the chiral intermediate, 1,1′-binaphthalene-2,2′-dicarbonitrile (**3**), is the key to directly integrate the chiral 1,1′-binaphthalene moiety into the central conjugated system of a subphthalocyanine analogue with help of the second species of achiral phthalonitrile^20^. Starting from commercially available chiral binaphthol (*R*/*S*)-**2**, 1,1′-binaphthalene-2,2′-dicarbonitrile (*R*/*S*)-**3** was obtained in a total yield of 21% by seven steps (Scheme S1 in Supporting Information). Subsequent cyclo-trimerization of (*R*/*S*)-**3** and tetrafluorophthalonitrile in the presence of boron tribromide (BBr_3_) in chlorobenzene at 60 °C, followed by treatment with 4-fluorophenol at 90 °C to displace the axial ligand, generated the target chiral subphthalocyanine analogue (*R*/*S*)-**1** in a yield of *ca.* 13%. As clearly demonstrated in Fig. 2, direct fusion of the chiral 1,1′-binaphthalene moiety into the central main conjugated chromophore of the subphthalocyanine analogue (*R*/*S*)-**1** renders this chiral compound intrinsically different structural feature from conventional chiral subphthtalocyanine derivatives with either inherent or induced chirality (Fig. 1).

This new chiral subphthalocyanine analogue gives satisfactory elemental analysis data. Its matrix assisted laser desorption ionization time-of-flight (MALDI-TOF) mass spectrum displays intense signals for both a molecular ion signal [M]^+^ and an axial-ligand-lost ion signal [M−OC_6_H_4_F]^+^ with the isotopic pattern closely resembling the simulated one (Figure S1 in Supporting Information). To further clarify its molecular structure and optical property, this subphthalocyanine analogue was characterized with different spectroscopic techniques including nuclear magnetic resonance (NMR), electronic absorption (UV-Vis absorption), circular dichroism (CD), and magnetic CD (MCD) spectroscopy.

**Figure 3:**
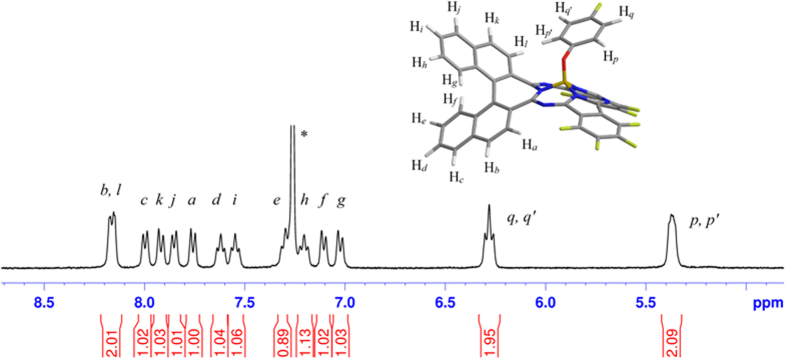
^1^H NMR spectrum with proton assignment of 1 recorded in CDCl_3_. The asterisk indicates the solvent impurity.

Figure 3 shows the ^1^H NMR spectrum of **1** recorded in CDCl_3_ at room temperature. Evidently, each of the binaphthyl protons exhibits a discrete signal due to the *C*_1_ molecular symmetry. With the help of ^1^H-^1^H COSY and NOESY measurements, all the protons are assigned in an unambiguous manner (Figures S2 and S3, and Table S1 in Supporting Information). The signals with chemical shift at *δ* = 8.16, 8.00, 7.76, 7.62, 7.31 and 7.11 ppm are attributed to a set of six protons on the inner side (*endo*) of the naphthene moiety bearing a higher deshielding effect, while the ones at *δ* = 8.16, 7.92, 7.85, 7.55, 7.20 and 7.02 ppm are assigned to the set of six protons on the outer side (*exo*) of naphthene moiety. It is worth noting that in the NOESY spectrum, two spatial couplings of H_*b*_-H_*c*_ and H_*j*_-H_*k*_ with distance of 2.43 Å for both are clearly found, confirming the above-mentioned assignment of protons. One can also notice from Fig. 3 that owing to the different induced ring current, every *endo* proton shows a larger chemical shift than its corresponding *exo* proton except for H_*l*_ (*exo*, *δ* = 8.16) and H_*a*_ (*endo*, *δ* = 7.76). The small shift for H_*l*_ and H_*a*_ is likely to originate from the additional deshielding effect from the free-rotating axial 4-fluorophenoxy group.

### Molecular structure

**Figure 4:**
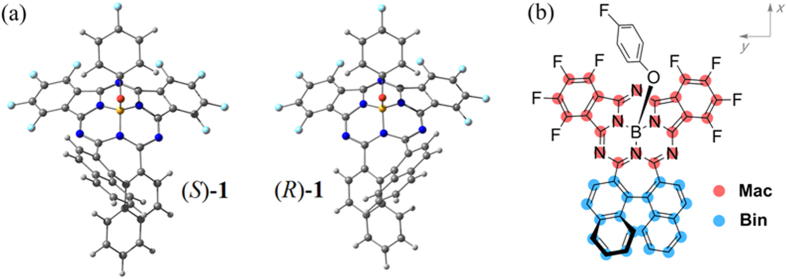
(**a**) Simulated molecular structures of (*S*)-**1** and (*R*)-**1** on basis of DFT calculations at M06/6-311G(d) level, and (**b**) scheme of molecular division into **Mac** and **Bin** groups.

Trials paid so far failed to produce single crystals of (*R*/*S*)-**1** suitable for X-ray diffraction analysis. As a consequence, the molecular structure of enantiomers (*R*/*S*)-**1** pair with typical chiral asymmetry, *C*_1_, was simulated on the basis of density function theory (DFT) calculations at M06/6-311G(d) level, and the corresponding result is summarized in Fig. 4(a)^21^. As shown in the calculation result (Fig. 4(b)), the length of the two C-C bonds that connect the central core-modified chromophore **Mac** and binaphthyl unit **Bin** is 1.48 Å, which locates just in the range of aromatic conjugated systems. This result manifests that these two moieties are integrated into the conjugated system in **1**. Actually, anisotropy of the induced current density (AICD) analysis confirms again that the π-electron delocalization region is extended over the whole molecular skeleton of **1** (Figure S5 in Supporting Information)^22^. Obviously, direct fusion of the intrinsic chiral segment, the binaphthyl unit, into the conjugated molecular structure of **1** endows this chiral subphthalocyanine analogue intrinsically different structural feature from conventional chiral conjugated systems, which do not contain chiral segment in the central conjugated chromophore.

**Figure 5:**
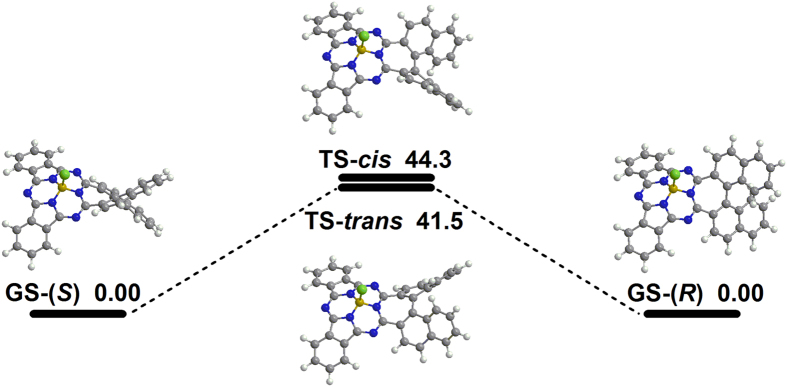
Configuration of ground states (GS) and transition states (TS) of **1** together with corresponding energy barrier (kcal · mol^−1^).

The chiral stability of this novel subphthalocyanine analogue is also uncovered by both DFT transition state calculations (Fig. 5) and chiral HPLC technique, Figure S7 (Supporting Information). DFT calculations for transition structures and internal reaction coordinate (IRC) paths (Figure S9, Supporting Information) show that the transition states of **1** exhibit *cis*- and *trans*-configurations with *C*_s_ molecular symmetry. The energy barriers of the two transition states resulting from the steric hindrance between two naphthene segment are up to 44.3 and 41.5 kcal·mol^−1^, respectively, which are difficult to be overcome below 200 °C. Such a large energy barrier guarantees enough thermo-stability for each enantiomer during the synthetic procedure. The enantiopurity of the final product (*R*)-**1** and (*S*)-**1** was confirmed by chiral HPLC (column: CHIRALPAK IA-3; eluent: propanol:hexane = 10:90) experiment result. As shown in the top of Figure S7 (Supporting Information), under the above-mentioned experimental condition the retention time for (*R*)-**1** and (*S*)-**1** amounts to 11.3 and 10.0 min, respectively. Nevertheless, no detective enantiomeric fraction could be found for either (*R*)-**1** or (*S*)-**1**, middle and bottom of Figure S7 (Supporting Information).

### Circular dichroism properties

**Figure 6:**
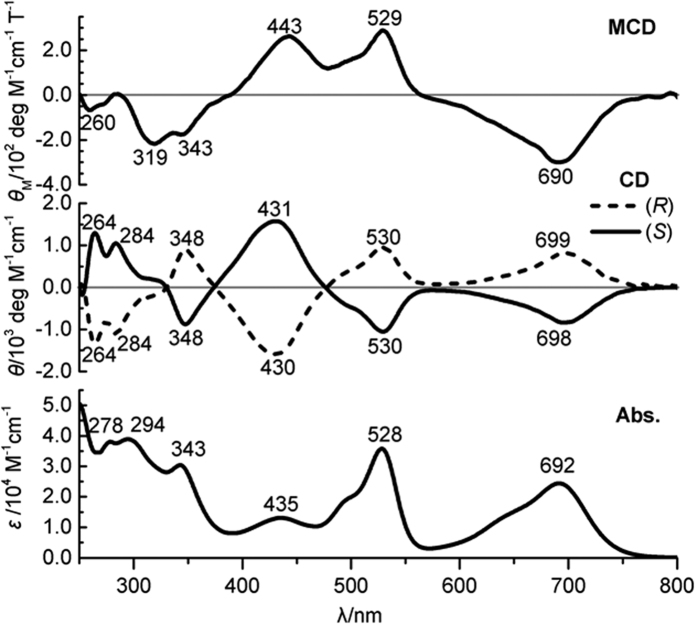
Electronic absorption, CD and MCD spectra of compound **1**.

The optical property of (*S*)-**1** and (*R*)-**1** is thoroughly characterized by the electronic absorption, CD and MCD spectra. In the electronic absorption spectrum, two absorption bands observed at 692 and 528 nm are attributed to the Q-bands (denoted as Q_1_- and Q_2_-band, respectively) of subphthlaocynine analogue with remarkable split resulting from the distorted molecular structure (bottom curve in Fig. 6)^20^. In addition, **1** has a weak absorption at 435 nm in the low-energy Soret band region, followed by an intense absorption at 343 nm belonging to the high-energy Soret band^18^,^23^. In the MCD spectrum (top curve in Fig. 6), a negative signal and a positive signal featured as two Faraday-*B* terms are observed at 690 and 529 nm in the Q-band region, indicating their homogeneous π-π^*^ transition nature with corresponding transitions localized on the main conjugated chromophore. The other two MCD peaks, a positive MCD signal at 443 nm in the low-energy Soret band region and a negative signal at 343 nm in the high-energy Soret band region, are also featured as Faraday-*B* terms. Finally, the optical activity of (*S*)-**1** and (*R*)-**1** pair is investigated by CD spectra (middle curves in Fig. 6). It is clear that the two isomers show perfect spectral mirror image over the entire region, confirming their enantiomeric molecular structures. In detail, along with the increased energy, (*S*)-**1** presents two negative CD signals at 698 and 530 nm in the Q-band region, a positive signal at 431 nm in the low-energy Soret band region, and again a negative signal at 348 nm in the high-energy Soret band region. In a similar way, the peaks at the same region with reverse signals are observed for (*R*)-**1**.

Significantly, (*S*)-(−)- or (*R*)-(+)-1,1′-binaphthyl substituent is able to induce negative and positive CD signals, respectively, throughout the entire absorption region of both phthalocyanine and subphthalocyanine compounds^5^,^15^,^24^. It is known that the induced CD intensity is proportional to *υ*_N_*υ*/(*υ*_N_^2^−*υ*^2^), where *υ*_N_ and *υ* are the absorption frequencies of binaphthyl group and subphthalocyanine chromophore, respectively^25^. In our case, (*S*)-**1** containing (*S*)-binaphthyl moiety yields two negative Q-band CD signals at 698 and 530 nm (*υ* = 0.43 × 10^15^ and 0.56 × 10^15^ s^−1^, respectively) with homogeneous π-π^*^ electron transitions, while the frequency of binaphthyl group *υ*_N_ that cannot be extracted from the absorption spectrum is estimated to be 0.93 × 10^15^ s^−1^ according to DFT calculation result (see the Methods section for details). As a result, the ratio of the CD intensity against *υ*_N_*υ*/(*υ*_N_^2^−*υ*^2^) for the Q_2_-band is calculated to be ~0.8 of that obtained for the Q_1_-band (Table S2 in Supporting Information). The quite close ratio value for these two Q-bands confirms that both Q-bands follow the mechanism of the induced optical activity from peripheral binaphthyl group^24^,^26^. (*R*)-**1** with (*R*)-binaphthyl moiety of two positive Q-band CD signals gives additional support for this point. As comparison, the ratio for the high-energy Soret band CD signal at 348 nm is only 0.07 of that obtained for the Q_1_-band, denying the same chirality origin. This result clearly implies the inherent rather than induced chirality characteristics for the high-energy Soret band of **1**. Moreover, the CD signal of the low-energy Soret band at 431 nm is opposite with respect to the two Q-band CD responses, also disclosing the inherent chirality nature.

To quantitatively evaluate the chiral dissymmetry of this novel chiral subphthalocyanine analogue, the anisotropic factor *g* for **1** was calculated according to Equation (1)^27^,^28^.
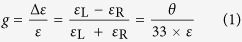
where *ε*_L_ and *ε*_R_ are the molar extinction coefficients of left and right circularly polarized light, respectively, while *θ* and *ε* the ellipticity and absorbance that are directly obtained from the experimental spectra. Table 1 compares the *g* values of the subphthalocyanine analogue containing chiral segment in the central chromophore with those of conventional chiral subphthalocyanine derivatives with either inherent chirality ((a)-3 in Fig. 1) or induced chirality (b) in Fig. 1. It is evident that the *g* factor for the CD signals in the Soret bands of **1** is dramatically enhanced to 3.7 × 10^−3^ compared with *ca*. 2.0 × 10^−4^ of the reference compound (a)-3 in Fig. 1, thanks to the formation of a helical conjugated molecular structure with great chiral dissymmetry^29^,^30^,^31^,^32^. On the other hand, the *g* factor for the CD signals of the Q-bands of **1** with induced chirality is also increased from *ca*. 6.1 × 10^−4^ of the reference compound (b) in Fig. 1 to 8.8 × 10^−4^ and 1.0 × 10^−3^, largely because of the shortened distance, *R*_*ij*_, between the chiral binaphthyl segment and the central conjugated chromophore of subphthalocyanine from *ca.* 9.2 Å for reference compound (b) (Fig. 1) to *ca.* 4.5 Å (measured from the simulated molecular structure in Fig. 4(a)). As demonstrated in the below Equation (3), the induced CD intensity is inversely proportional to the cube of the distance between the central conjugated chromophore and the chiral group, i.e., *θ* ∝ *R*_*ij*_^−3^, which is responsible for the increased *g* factor of the CD signal of the Q-bands of **1**. Considering such a fact that the Q-band CD signal of the reference compound is induced by three binaphthyl groups, while that of **1** by only one binaphthyl segment, the increase in the *g* factor from reference compound to **1** as detailed above clearly indicates the significant effect of the direct fusion of the binaphthyl segment into the central subphthalocyanine chromophore on the molecular dissymmetry of conjugated systems.

### Electronic structure

On the basis of the quantum mechanics, the optical activity for chiral molecular compounds could be described by rotational strength, *R*:
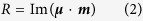


For the conjugated systems with inherent chirality, both ***μ*** and ***m*** are delocalized over the whole π system. In contrast, ***μ*** and ***m*** are decomposed to the contribution from either the main chromophore or additional chiral substituent(s) for the conjugated systems with induced chirality, thus rendering an alternative way to calculate their rotational strength:
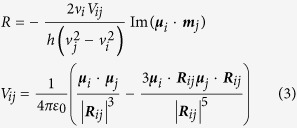
in which subscripts *i* refers to the main conjugated chromophore with electric dipole-allowed transition moment ***μ***_*i*_ at energy *hν*_*i*_, *j* represents the perturbation moieties [the additional chiral substituent(s)] in the molecule with electric dipole-allowed transition moment***μ***_*j*_ and magnetic dipole-allowed transition moment ***m***_*j*_ (including both intrinsic and gauge-dependent external magnetic dipole moment) at energy *hν*_*j*_, ***R***_*ij*_ stands for the distance between groups *i* and *j*, and *V*_*ij*_ is the Coloumb interaction coupling between the two moieties^33^,^34^. As a result, a CD signal of the main conjugated chromophore with local electric dipole moment can be induced by coupling with the magnetic dipole moment of an external chiral group, leading to the original concept of induced optical activity. It should be pointed out that the existence of only Coulomb interaction between the main conjugated chromophore and additional chiral group(s) defines the essential feature of conjugated systems with induced chirality, while sufficient orbital correlation between neighboring components throughout the whole molecular skeleton determines the conjugated systems with inherent chirality nature.

**Figure 7:**
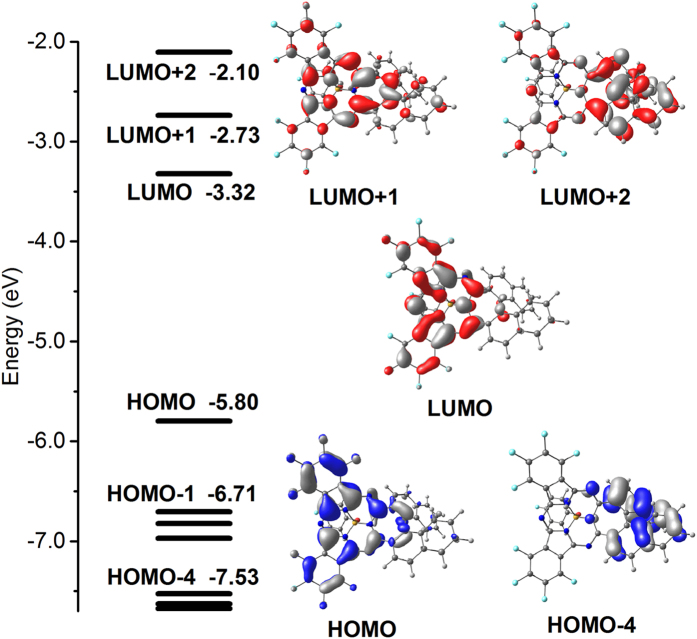
Diagram of frontier molecular orbitals of (*S*)-**1**.

**Figure 8:**
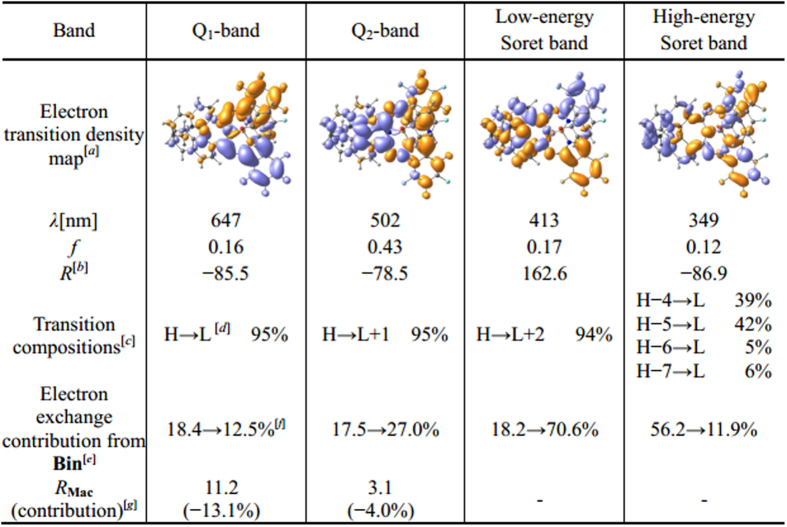
Calculated electronic transition density maps, excited wavelengths (*λ*), oscillator strengths (*f* ), rotational strengths (*R*), and orbital compositions of important transitions of (***S***)-**1**. [*a*] The isovalue of electron transition density map is 0.008. [*b*] The unit is 10^−40^ erg-esu-cm/Gauss. [*c*] The transition compositions with more than 5% contribution are listed. [*d*] H refers to HOMO and L to LUMO. [*e*] Transition contribution of each fragment is summation of composed orbital contributions of each fragment. Orbital compositions more than 0.001% are counted. [*f* ] From ground state to excited state. [*g*] Rotatory strength contribution is decomposed to **Mac** and **Bin** segments *via* Mulliken charge.

Towards deep understanding of the spectral properties and chirality nature of this novel chiral subphthalocyanine analogue, DFT and time-dependent DFT (TDDFT) calculations have been performed at the M06/6-311G(d) level. Frontier molecular orbital (MO) patterns and their energy levels are illustrated in Fig. 7. Generally, HOMO, LUMO and LUMO + 1 mainly distribute on the subPc macrocycle, as highlighted in red in Fig. 4(b); while HOMO − 4 and LUMO + 2 mainly reside on the binaphthyl unit, as highlighted in blue in Fig. 4(b). Therefore, it is reasonable to divide the whole molecule into the main conjugated chromophore (**Mac**) segment and peripheral binaphthyl (**Bin**) segment. Figure 8 summarizes the TDDFT calculation results with the help of the electron density difference maps and fragment contributions. As can be seen, the Q_1_-band absorption estimated at 647 nm is contributed almost exclusively by the transition HOMO → LUMO, which localizes mainly on the **Mac** moiety of 81.6 → 87.5% scale with only 18.4 → 12.5% electron exchange from **Bin**. Similarly, the Q_2_-band absorption estimated at 502 nm is dominantly contributed by the transition HOMO → LUMO + 1 with the **Mac** moiety of 82.5 → 73.0%, suggesting a weak electron exchange from **Bin** with 17.5 → 27.0% in addition to the Coulomb interaction. The weak electron transition in the low-energy Soret band estimated at 413 nm is contributed by HOMO → LUMO + 2 with participation from the **Bin** part of 18.2 → 70.6%, indicating an intramolecular transfer from **Mac** to **Bin** with sufficient electron correlation between these two moieties. The high-energy Soret band is composed by many weak transitions, among which the strongest transition contributed from inner occupied orbitals to LUMO calculated at 349 nm might correspond to the observed absorption at 343 nm^18^,^35^. Interestingly enough, an intramolecular transfer from **Bin** to **Mac** with contribution from the **Bin** moiety of 56.2 → 11.8% is involved in this transition, also suggesting the sufficient electron exchange between these two moieties. Evidently, the two Q-band electron transitions are actually contributed dominantly by the **Mac** moiety with up to 84.6 and 77.8% on average, respectively, indicating the dominant Coulomb interaction between **Mac** and **Bin** and in turn disclosing the dominantly induced chirality characteristics for these two Q-band CD responses of **1**. This is in good agreement with the CD spectroscopic analysis as detailed above. However, this is not the case for both the low-energy Soret and high-energy Soret absorption bands. As indicated above, **Mac** moiety contributes 29.4% only to the excited state of the transition that is responsible for the low-energy Soret band, while the ground state of the transition responsible for the high-energy Soret band is contributed by 43.8% only from the **Mac** moiety, revealing the significant orbital correlation between **Mac** and **Bin** for these two Soret bands of **1**. This in turn confirms the inherent chirality characteristics of the two Soret-band transitions.

In addition, the induced chirality nature of the two Q-bands might be further revealed by CD contribution analysis with help of transition dipole moment decomposition, by which both the electric and magnetic transition dipole moments can be quantitatively decomposed into the contribution from group **Mac** and **Bin** (Fig. 4(b) and Table S3 in Supporting Information). The intrinsic rotational strength of **Mac** and **Bin** is obtained by direct scalar multiplication of their respective electric and magnetic transition dipole moments. As listed in Fig. 8, the rotational strength provided by **Mac** is calculated to amount to only −13.1% for the Q_1_-band and −4.0% for the Q_2_-band, respectively, with the rest totally originating from **Bin** as well as its interaction with **Mac**. Obviously, the **Mac** chromophore yields two Q-band CD signals largely caused by **Bin**. This confirms, again, the dominant induced chirality characteristics for the two Q-band CD signals. As comparison, the electron transitions that are responsible for both the low- and high-energy Soret bands spread over both **Mac** and **Bin** moieties, resulting in inapplicability of the transition dipole moment decomposition analysis for these two band transitions. Altogether, this novel subphthalocyanine analogue exhibits optical activity with both induced and inherent chirality nature, thus representing the first example of subphthalocyanine with mixed chirality characteristics.

## Conclusion

In summary, chiral 1,1′-binaphthalene moiety has been directly fused into the conjugated skeleton of a subphthalocyanine analogue, resulting in an unprecedented chiral segment-containing π-conjugation system with improved chiral dissymmetry. Notably, this unconventional chiral subphthalocyanine analogue integrates inherent and induced chirality, which is significantly distinct from the conventional chiral subphthalocyanine derivatives only showing either inherent or induced chirality nature. As a result, this type of chiral subphthalocyanine analogue exhibits the significantly enhanced optical activity compared with conventional chiral subphthalocyanines, and furthermore the origin of such improved optical activity is explored in detail by both experiment and theory. This work not only represents a brand new type of chiral conjugated system for device application, but also establishes the guideline towards rational design of chiral molecular structures with giant optical activity.

## Methods

### General remarks

Tetrahydrofuran (THF), triethylamine, chlorobenzene, and dichloromethane were freshly distilled from CaH_2_ under nitrogen. Column chromatography was carried out on silica gel (Merck, Kieselgel 60, 70–230 mesh) columns with the indicated eluents. The electrolyte [Bu_4_N][ClO_4_] was recrystallized twice from acetone. All other reagents and solvents were of commercial reagent grade and used as received. NMR spectra were recorded on a Bruker DPX 400 spectrometer in indicated solvent, using residual CHCl_3_ solvent resonance as an internal reference for ^1^H (*δ* = 7.26 ppm) and BF_3_∙OEt_2_ as an external reference for ^11^B (*δ* = 0.00 ppm). Elemental analyses were performed on an Elementar Vavio El III. HPLC was performed on a LC-9210NEXT instrument of JAI Co., Ltd. equipped with a preparative CHIRALPAK IA-3 column (250 mm) by monitoring absorbance at 528 nm. Electronic absorption spectra were recorded on a Hitachi U-4100 spectrophotometer. CD and MCD spectra were recorded on a JASCO J-1500 spectropolarimeter equipped with a JASCO electromagnet which produced both parallel and anti-parallel magnetic fields of 1.60 T. IR spectra were recorded in KBr pellets with 2 cm^−1^ resolution using a Bruker Tensor 37 spectrometer. MALDI-TOF mass spectra were taken on a Bruker BIFLEX III ultrahigh resolution Fourier transform ion cyclotron resonance (FT-ICR) mass spectrometer with alpha-cyano-4-hydroxycinnamic acid as matrix. Electrochemical measurements were carried out with a BAS CV-50W voltammetric analyzer. The cell comprised inlets for a glassy carbon disk working electrode of 2.0 mm in diameter and a silver-wire counter electrode. The reference electrode was Ag/Ag^+^, which was connected to the solution by a Luggin capillary whose tip was placed close to the working electrode. It was corrected for junction potentials by being referenced internally to the ferrocenium/ferrocene (Fc^+^/Fc) couple [E_1/2_ (Fc^+^/Fc) = 501 mV *vs.* SCE]. Typically, a 0.1 mol dm^−3^ solution of [Bu_4_N][ClO_4_] in CH_2_Cl_2_ containing 0.5 mmol dm^−3^ of sample was purged with nitrogen for 10 min, then the voltammograms were recorded at ambient temperature. The scan rate was 75 mV∙s^−1^ for cyclic voltammetry measurement.

### Preparation of (*R*/*S*)-1

To a chlorobenzene (0.5 mL) solution of **3** (76.0 mg, 0.25 mmol) in a 10 mL reactor was added boron tribromide (1.0 M in CH_2_Cl_2_, 0.5 mL, 0.5 mmol) and heated at 60 °C for 2 min. Keeping the temperature, a chlorobenzene (0.5 mL) solution of 3,4,5,6-tetraflourophthalonitrile (120 mg, 0.60 mmol, 2.4 eq.) was added dropwise into the reactor, and the reaction mixture was continually stirred for 2–4 hours until the starting material **3** was completely consumed. After removal of the solvent under vacuum, the residue was soon dealt with several drops of anhydrous THF (0.2 mL) and chromatographed on silica gel column (hexane: toluene = 2: 3) to remove impurities. The collected intermediate product was further treated with 4-fluorophenol (150 mg, excess amount) at 90 °C for 2 h. Repeated chromatography (hexane: toluene = 2: 3) followed by recrystallization from CHCl_3_ and hexane afforded the final enatiopure product **1** (26.0 mg, 0.032 mmol, 13%).

### Computational details

M06 method with 6-311G(d) basis set was used in all calculations and carried out by *Gaussian 09* (*Version D.01*) program^36^. Wavefunction analyses including orbital composition analysis, electron transition density analysis, and transition dipole moment decomposition were processed by *Multiwfn* (*Version 3.3.8*) program^37^. Calculation for the absorption frequencies of binaphthyl group (*υ*_N_): The conformation of binaphthyl group under calculation is the same as that of **Bin** moiety in geometry-optimized molecule **1** and saturated by hydrogen (Fig. 2). Through TDDFT calculation, the first excited state was estimated to locate at 324 nm with large oscillator strength (*f* = 0.21). The conversion from absorption wavelength (*λ*) to frequency (*υ*) followed *υ* = *c*_0_/*λ*, where *c*_0_ is the light speed. As a result, the corresponding absorption frequency *υ*_N_ for the first excited state was 0.93 × 10^15^ s^−1^. It is worth noting that the calculated *υ*_N_ was an estimated value that is qualitatively valid for calculating the factor *υ*_N_*υ*/(*υ*_N_^2^−*υ*^2^) (Table S2 in Supporting Information) and judging the chirality mechanism.

## Additional Information

**How to cite this article**: Zhao, L. *et al*. Integration of inherent and induced chirality into subphthalocyanine analogue. *Sci. Rep.* **6**, 28026; doi: 10.1038/srep28026 (2016).

## Supplementary Material

Supplementary Information

## Figures and Tables

**Table 1:** Comparison of anisotropic factor, *g*, of **1** with that if two conventional chiral subphthalocyanine derivatives reported previously.

**Compound**	**Absorption band**	**Abs. (10**^**4**^ ***ε***)	**Inherent CD (10**^**3**^ ***θ***)	**Induced CD (10**^**3**^ ***θ***)	**10**^−**3**^ ***g***
**1**	Q_1_	2.4	–	0.83	1.0
	Q_2_	3.6		1.05	0.88
	l-Soret	1.3	1.58	–	3.7
(b)^[*a*]^	Q	10.0	–	2.01	0.61
(a)-3^[*a*]^	l-Soret	3.8	0.25	–	0.20

[*a*] Shown in Fig. 1.
