# Supplementary material for: Integration of inherent and induced chirality into subphthalocyanine analogue
Source: Sci Rep. 2016 Jun 13;6:28026. doi: 10.1038/srep28026 (PMC4904797; doi:10.1038/srep28026)
Supplement: Supplementary Information [file srep28026-s1.doc]

Supporting Information

# Integration of inherent and induced chirality into subphthalocyanine analogue

**Luyang Zhao,^†^ Dongdong Qi,^†^ Kang Wang,^†^ Tianyu Wang,^†^ Bing Han,^‡^ Zhiyong Tang,^‡^* and Jianzhuang Jiang^†^***

Caption of Contents

**Synthetic procedure**

**Electrochemical properties**

**Scheme S1.** Detailed synthetic procedure from commercially available **2** to the precursor **3**. (i) Tf_2_O, CH_2_Cl_2_, Et_3_N, −78→0°C, 2 h; (ii) CH_3_MgBr, Ni(dppp)Cl_2_, Et_2_O, 0°C, overnight; (iii) *N*-bromosuccinimide, benzoyl peroxide, CCl_4_, 80°C, 1 h; (iv) AgNO_3_, THF/H_2_O, 85°C, 1 h; (v) NaClO_2_, H_2_O_2_, CH_3_CN/H_2_O, 50°C, 1 h; (vi) SOCl_2_, 80°C, 1 h; THF, NH_3_, 0°C, 1 h; (vii) Tf_2_O, CH_2_Cl_2_, Et_3_N, 0°C, 2 h.

**Figure S1.** MALDI-TOF mass spectrum of **1**.

**Figure S2.** COSY ^1^H-^1^H NMR spectrum of **1** in the range of *δ* 6.9-8.3 ppm recorded in CDCl_3_.

**Figure S3.** NOESY ^1^H-^1^H NMR spectrum of **1** in the range of *δ* 6.9-8.3 ppm recorded in CDCl_3_.

**Figure S4.** ^13^C NMR spectrum of **1** recorded in CDCl_3_. The asterisk indicates solvent impurity.

**Figure S5.** IR spectrum of **1** in the region of 400-2000 cm^−1^ with 2 cm^−1^ resolution.

**Figure S6.** AICD plot at isovalue of 0.05 of (*S*)-**1** (bottom view).

**Figure S7.** HPLC chromatogram of a random mixture of (*R*)-**1**/(*S*)-**1** (top), (*R*)-**1** (middle), and (*S*)-**1** (bottom) using a preparative CHIRALPAK IA-3 column.

**Figure S8.** Cyclic voltammogram and differential pulse voltammogram of **1** in CH_2_Cl_2_ containing 0.1 M [NBu_4_][ClO_4_] at scan rate of 75 mV⋅s^−1^.

**Figure S9.** IRC paths of transition structures mentioned in Figure 5.

**Figures of ^1^H and ^13^C NMR spectra of compounds 3-7**

**Table S1.** ^1^H NMR data (*δ*) for **1** recorded in CDCl_3_ at 25°C.

**Table S2.** Calculation of ratio of CD intensity against coefficient factor *υ*_N_*υ*/(*υ*_N_^2^−*υ*^2^).

**Table S3.** Electric transition dipole moment ***μ*** and magnetic transition dipole moment ***m*** decomposition into **Mac** and **Bin** group contributions. All the listed values are in atomic unit.

**Z-matrices of optimized structures**

**References for supporting information**

**Synthetic procedure**

***Preparation of (R/S)-1,1'-binaphthalene-2,2'-diyl bis(trifluoromethanesulfonate) (4):***^[1]^ A single necked flask charged with a solution of (*R*/*S*)-BINOL (**3**) (14.3 g, 50.0 mmol) and Et_3_N (15.2 g, 20.9 mL, 150 mmol) in CH_2_Cl_2_ (125 mL) was cooled to −78°C, and Tf_2_O (31.0 g, 18.5 mL, 110 mmol) was added dropwise. Then the resulting mixture was warmed to 0°C for 2 h, and sequentially washed by 1.0 M HCl aq., saturated NaHCO_3_ aq., brine, and water. The organic phase was dried over anhydrous Na_2_SO_4_, filtered, and concentrated to afford the desired product **4** as orange solid (27.5 g, 50.0 mmol, 99% yield), which was used for the next step without further purification. ^1^H NMR (400 MHz, CDCl_3_, 298 K): *δ* = 8.15 (d, 2H), 8.01 (d, 2H), 7.61 (m, 4H), 7.42 (t, 2H), and 7.26 (d, 2H). ^13^C NMR (400 MHz, CDCl_3_, 298 K): *δ* = 145.39, 133.15, 132.36, 132.01, 128.35, 128.12, 127.83, 127.41, 126.92, 126.59, 123.44, 119.73, 119.24, and 116.55.

***Preparation of mixed (R/S)-2,2'-dimethyl-1,1'-binaphthalene (5):***^[1]^ To a solution of **4** (27.5 g, 50.0 mmol) and Ni(dppp)Cl_2_ (2.7 g, 5.0 mmol) in ether (200 mL) was added methyl magnesiumbromide (3.0 M, 50 mL, 150 mmol) dropwise at 0°C. Then the reaction mixture was heated to reflux overnight, and was quenched by cautious addition of diluted with 5% HCl aq. (50 mL). The organic layer was washed sequentially with NaHCO_3_ aq., brine and water, dried over anhydrous Na_2_SO_4_ and concentrated to afford **5** as dark yellow solid (11.5g, 40.4 mmol, 81% yield), which was used for the next step without further purification. ^1^H NMR (400 MHz, CDCl_3_, 298 K): *δ* = 7.89 (t, 2H), 7.52 (d, 2H), 7.40 (t, 2H), 7.21 (t, 2H), 7.05 (d, 2H), and 2.04 (s, 3H). ^13^C NMR (400 MHz, CDCl_3_, 298 K): *δ* = 135.17, 134.31, 132.81, 132.26, 128.86, 128.66, 127.98, 127.95, 127.49, 127.45, 126.28, 125.96, 125.52, 125.05, 124.81, 20.15, 19.98, and 19.82.

***Preparation of (R/S)-2,2'-bis(dibromomethyl)-1,1'-binaphthalene (6):***^[2]^ To a 1 L flask was added **5** (11.5 g, 40.7 mmol) dissolved in CCl_4_ (150 mL). To this solution was added *N*-bromosuccinimide (58.0 g, 320 mmol) and benzoyl peroxide (0.50 g, 2.1 mmol). The reaction was refluxed for 18 h. After cooling down to room temperature, filtration and removal of the solvent under vacuum afforded a residue which was then taken up in CH_2_Cl_2_ (200 mL) and H_2_O (200 ml). The aqueous phase was separated and further extracted with CH_2_Cl_2_. The combined organic layers were washed with a saturated aqueous Na_2_CO_3_ aq., brine and water. The organic phase was dried over Na_2_SO_4_, filtered and the solvent was removed under vacuum to provide crude **6** (24.0 g, 40.0 mmol, 98.1% yield) as yellow solid. ^1^H NMR (400 MHz, CDCl_3_, 298 K): *δ* = 8.24 (d, 2H), 8.15 (d, 2H), 7.96 (d, 2H), 7.54 (t, 2H), 7.30 (t, 2H), 6.98 (d, 2H), and 6.21 (s, 2H). ^13^C NMR (400 MHz, CDCl_3_, 298 K): *δ* = 138.33, 133.82, 130.89, 130.77, 128.30, 128.04, 127.93, 127.83, 127.48, 126.57, 126.49, and 39.14.

***Preparation of (R/S)-1,1'-binaphthalene-2,2'-dicarboxylic acid (7):***^[3]^ To a solution of crude **6** (2.99 g, 5.00 mmol) in THF (40 mL) was added AgNO_3_(8.5 g, 50 mmol) and water (20 mL), and the mixture was heated at reflux under nitrogen for 1 h. The solution was allowed to cool to room temperature, to which was added brine. Ag salt precipitate was filtered off and the filtrate was extracted with Et_2_O, and the combined organic phase was washed with brine and dried over anhydrous Na_2_SO_4_. The solvent was evaporated and the residue was purified by silica gel column chromatography (hexane: ethyl acetate = 20:1) to provide crude (*R*/*S*)-1,1'-binaphthalene-2,2'-dicarbaldehyde as thick orange liquid. Then this crude product (1.03 g, 3.31 mmol), 30 % aqueous hydrogen peroxide (0.73 mL, 7.2 mmol), sodium chlorite (1.0 g, 11 mmol) sodium phosphate monobasic monohydrate (0.27 g, 2.0 mmol) were dissolved in a mixture of water (10 mL) and acetonitrile (20 mL) and the mixture was stirred for 1 h at 50°C. After cooling down to room temperature, the reaction mixture was dealt with sodium sulfite (0.5 g) and then 2.0 M HCl aq. (20 mL), and extracted by ethyl acetate. The organic phase was dried by anhydrous Na_2_SO_4_ and concentrated. The resulting solid was recrystallized by CHCl_3_/hexane to afford the desired product **8** (1.06 g, 3.10 mmol, 93.7% yield from **6**) as light yellow solid. ^1^H NMR (400 MHz, CDCl_3_, 298 K): *δ* = 8.10 (d, 2H), 7.92 (m, 4H), 7.47 (t, 2H), 7.13 (t, 2H), and 6.88 (d, 2H). ^13^C NMR (400 MHz, CDCl_3_, 298 K): *δ* = 172.04, 141.73, 135.34, 132.82, 128.07, 127.83, 127.22, 126.76, 126.46, and 125.53.

***Preparation of (R/S)-1,1'-binaphthalene-2,2'-dicarbonitrile (3):***^[4]^ A solution of **8** (3.42 g, 10.0 mmol) in thionyl chloride (10 ml) was refluxed with stirring for 1 h. After evaporation of solvent, the residue was dissolved by anhydrous THF (5.0 ml), with NH_3_ gas bubbling for 1 h. Then the mixture was poured into 10% HCl aq., and was extracted with CH_2_Cl_2_ (100 ml). The organic layer was washed with brine and water and concentrated to provide crude (*R*/*S*)-1,1'-binaphthalene-2,2'-dicarboxamide. To this crude product (680 mg, 0.20 mmol) was dissolved in the mixture of CH_2_Cl_2_ (2 mL) and Et_3_N (1.1 ml), and was titrated with Tf_2_O (0.8 mL) at 0°C. Then the mixture was heated to 40°C for 1 h. After removal of solvent, the residue was dissolved in CH_2_Cl_2_ and washed by 1 M HCl aq., saturated NaHCO_3_, brine, and water, and dried by anhydrous Na_2_SO_4_. Concentration and column chromatography (hexane: CH_2_Cl_2_ = 1: 2) afforded the desired enantiopure **3** (280 mg, 0.92 mmol, 46.1% yield) as light yellow solid powder. ^1^H NMR (400 MHz, CDCl_3_, 298 K): *δ* = 8.14 (d, 2H), 8.04 (d, 2H), 7.85 (d, 2H), 7.67 (t, 2H), 7.44 (t, 2H), and 7.17 (d, 2H). ^13^C NMR (400 MHz, CDCl_3_, 298 K): *δ* = 140.63, 134.98, 131.89, 130.50, 129.38, 128.82, 128.61, 126.50, 117.61, and 111.67.

***Characterizations of (R/S)-1:*** *s*, m.p. > 300°C. ^1^H NMR (400 MHz, CDCl_3_, 298 K): *δ* = 8.16 (d, 2H), 8.00 (d, 1H), 7.92 (d, 1H), 7.85 (d, 1H), 7.76 (d, 1H), 7.62 (t, 1H), 7.55 (t, 1H), 7.31 (t, 1H), 7.20 (t, 1H), 7.11 (d, 1H), 7.02 (d, 1H), 6.28 (t, 2H), and 5.37 (m, 2H). ^13^C NMR(400 MHz, CDCl_3_, 298 K): *δ* = 159.76, 159.28, 147.97, 143.03, 142.80, 134.78, 134.71, 134.10, 134.06, 133.90, 131.85, 131.36, 130.92, 129.33, 128.86, 128.19, 128.04, 127.90, 127.42, 126.98, 126.67, 120.42, 120.34, 115.72, 115.49. ^11^B NMR (400 MHz, CDCl_3_, 298 K): *δ* = −12.1 (s, 1B). UV-vis (CHCl_3_): *λ*_max_ [nm] (*ε*) = 692 (24 400), 528 (35 800), 435 (13 100), 343 (30 400), 294 (38 900), 278 (38 100). MS (MALDI-TOF): *m/z* 714.828 (Calcd for [M−OC_6_H_4_F]^+^ 715.109), 825.877 (Calcd. for [M]^+^ 826.134). Anal. Calcd. for C_44_H_16_BN_6_OF_9_ (%): C, 63.95; H, 1.95; N, 10.17. Found: C, 63.92; H, 2.47; N, 9.79. IR (film): 1597, 1413, 1385, 1092, and 1076 cm^−1^.

***Electrochemical properties.*** The redox behavior of **1** was studied by cyclic voltammetry (CV) and differential pulse voltammetry (DPV). As shown in Figure S4, the chiral subphthalocyanine analogue **1** exhibited one quasi-reversible one-electron oxidation (1.02 V *vs.* SCE) and two quasi-reversible one-electron reductions (−0.82 and −0.56 V *vs.* SCE). The potential difference between the first oxidation and the first reduction, which corresponded the HOMO-LUMO gap, was 1.58 V for **1**, much smaller than that for conventional subphthalocyanine derivatives, indicating the extended conjugated system of this compound due to the conjugation between **Mac** and **Bin** moieties.


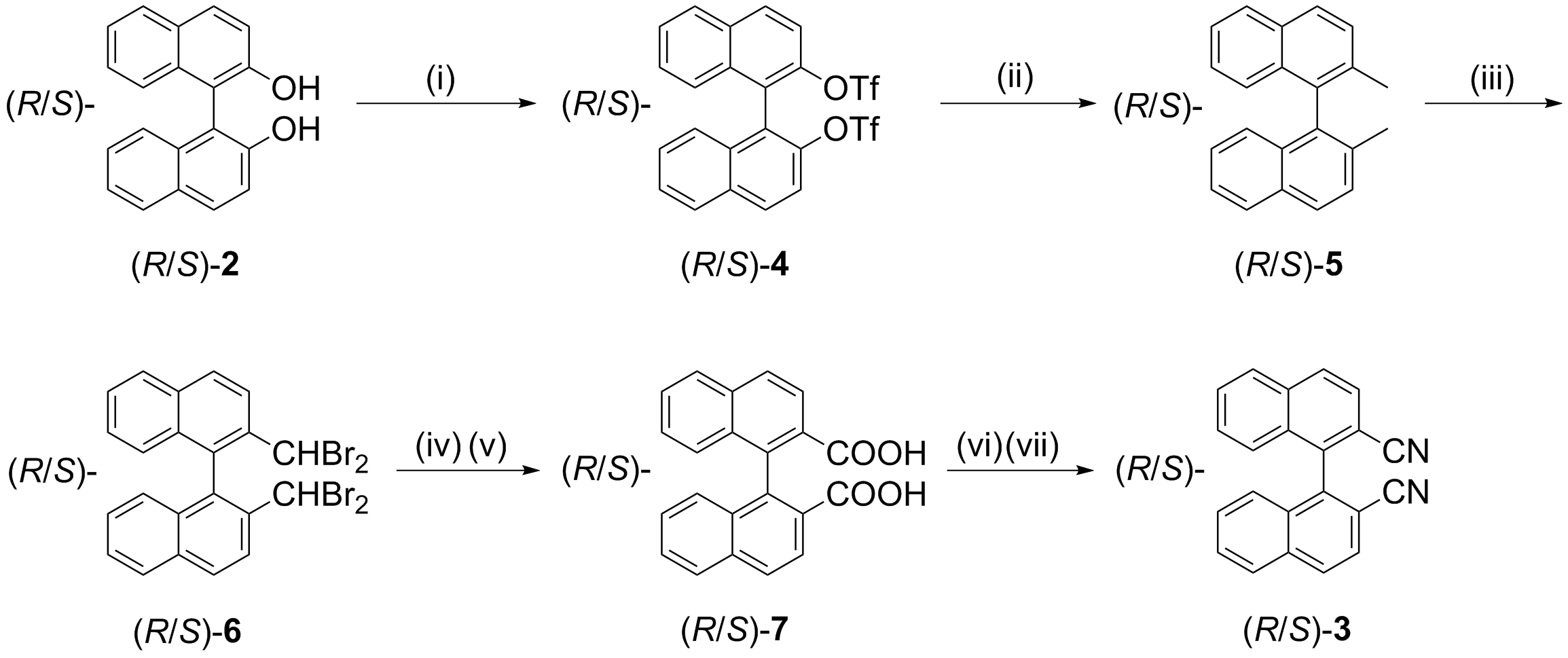


**Scheme S1.** Detailed synthetic procedure from commercially available **2** to the precursor **3**. (i) Tf_2_O, CH_2_Cl_2_, Et_3_N, −78→0°C, 2 h; (ii) CH_3_MgBr, Ni(dppp)Cl_2_, Et_2_O, 0°C, overnight; (iii) *N*-bromosuccinimide, benzoyl peroxide, CCl_4_, 80°C, 1 h; (iv) AgNO_3_, THF/H_2_O, 85°C, 1 h; (v) NaClO_2_, H_2_O_2_, CH_3_CN/H_2_O, 50°C, 1 h; (vi) SOCl_2_, 80°C, 1 h; THF, NH_3_, 0°C, 1 h; (vii) Tf_2_O, CH_2_Cl_2_, Et_3_N, 0°C, 2 h.


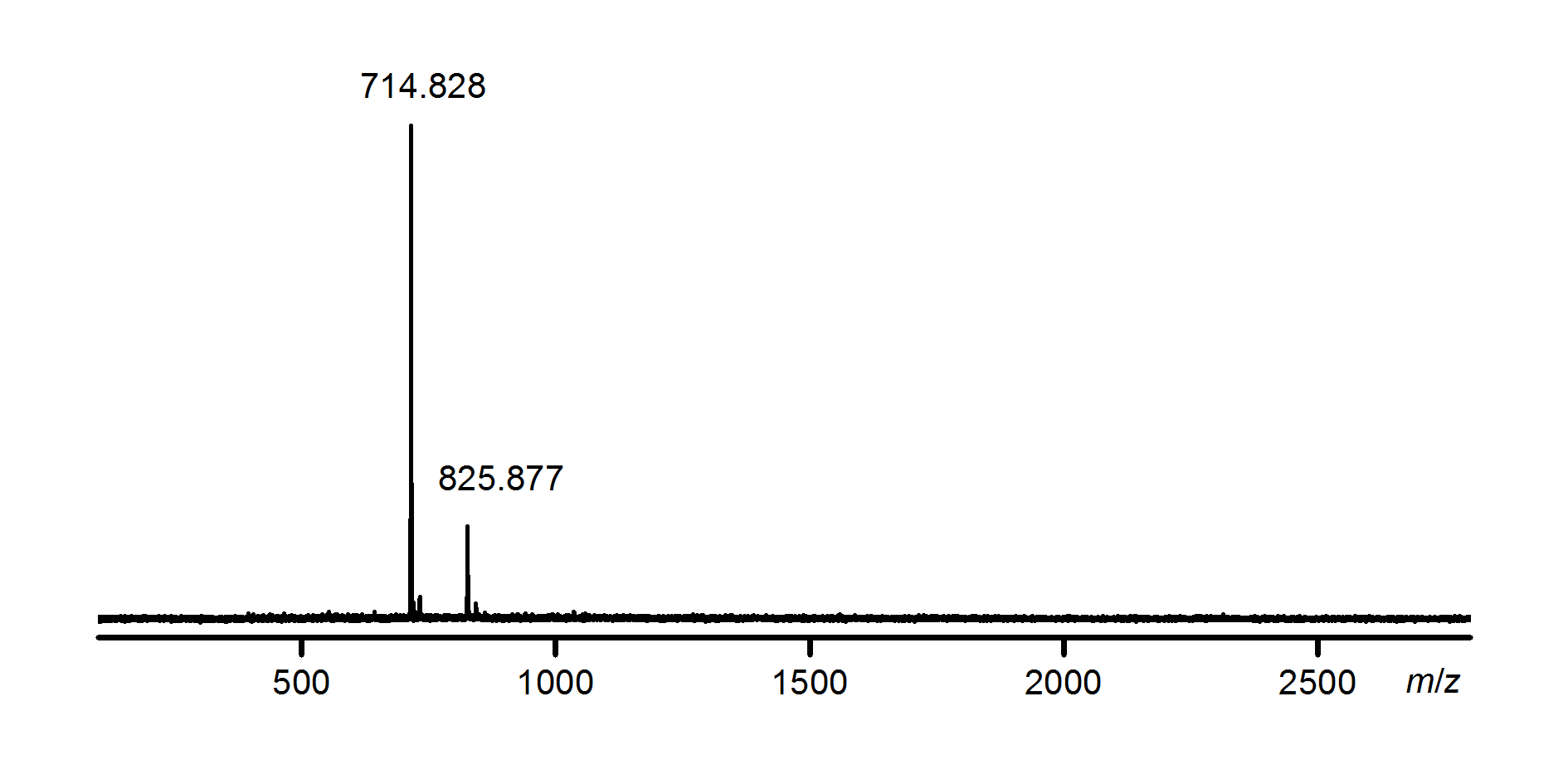


**Figure S1.** MALDI-TOF mass spectrum of **1**.


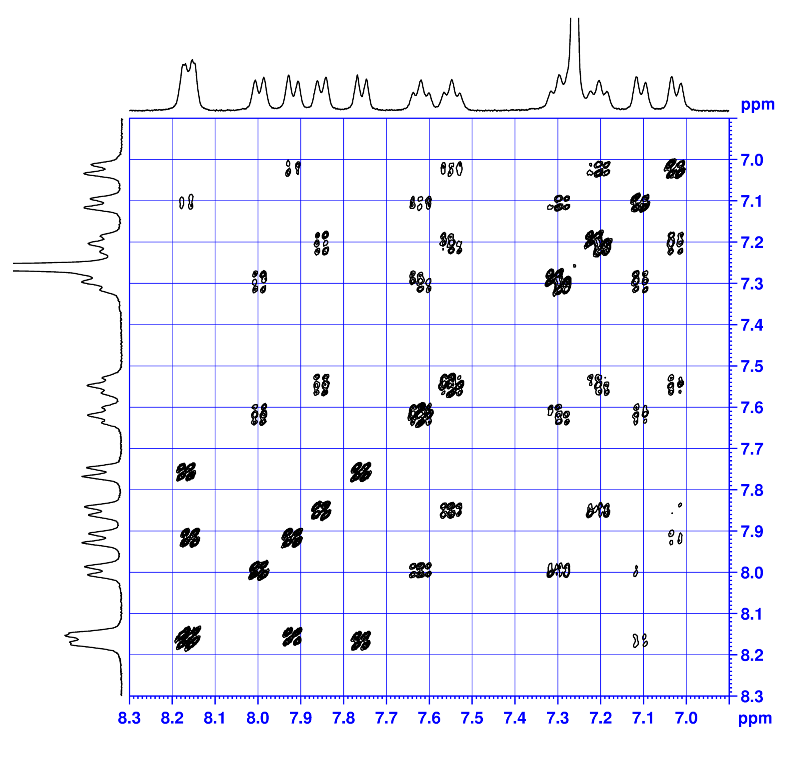


**Figure S2.** COSY ^1^H-^1^H NMR spectrum of **1** in the range of *δ* 6.9-8.3 ppm recorded in CDCl_3_.


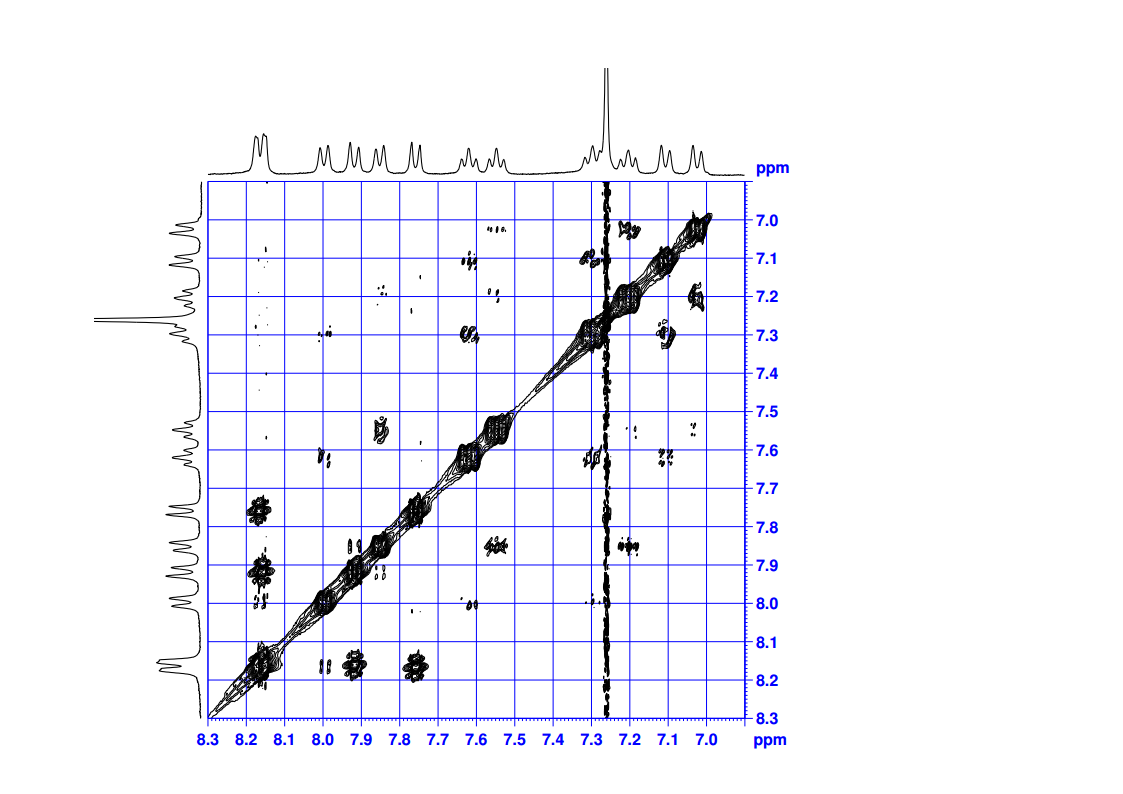


**Figure S3.** NOESY ^1^H-^1^H NMR spectrum of **1** in the range of *δ* 6.9-8.3 ppm recorded in CDCl_3_.


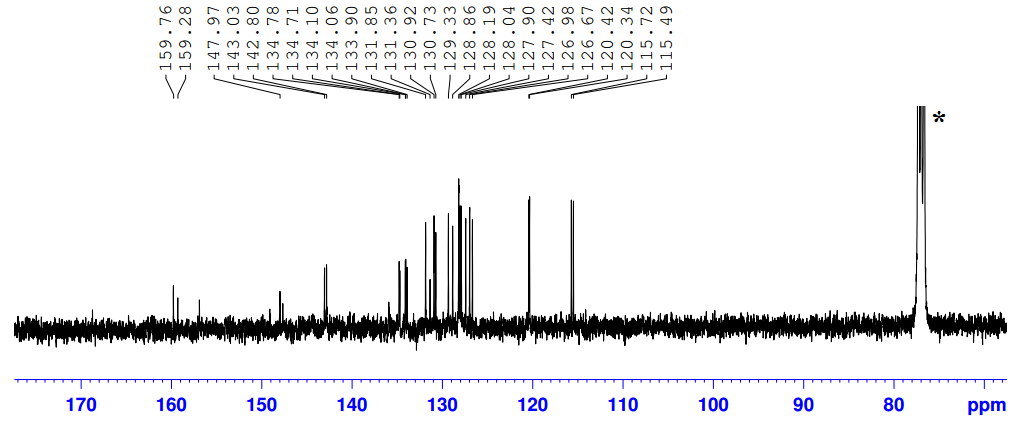


**Figure S4.** ^13^C NMR spectrum of **1** recorded in CDCl_3_. The asterisk indicates solvent impurity.


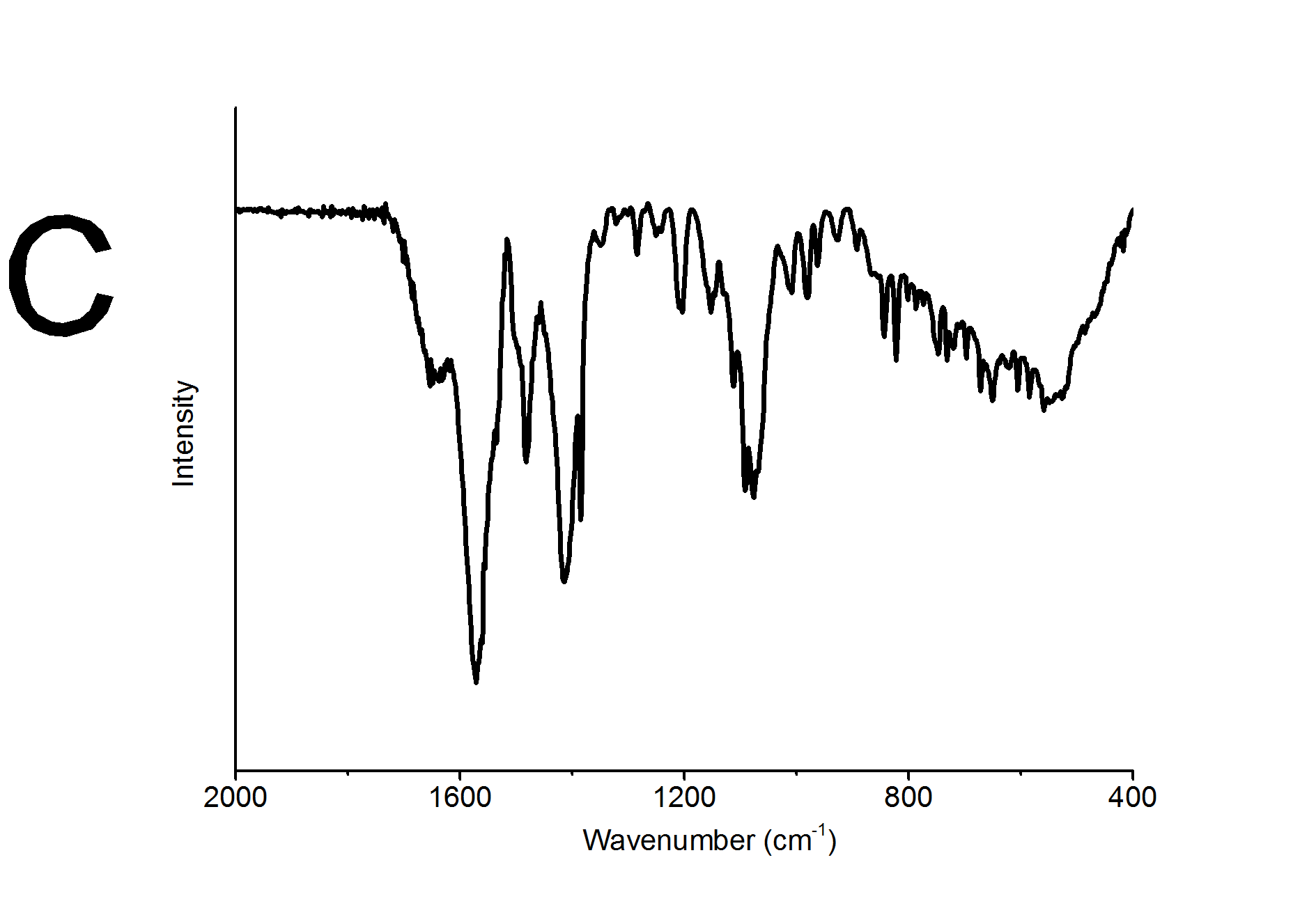


**Figure S5.** IR spectrum of **1** in the region of 400-2000 cm^−1^ with 2 cm^−1^ resolution.


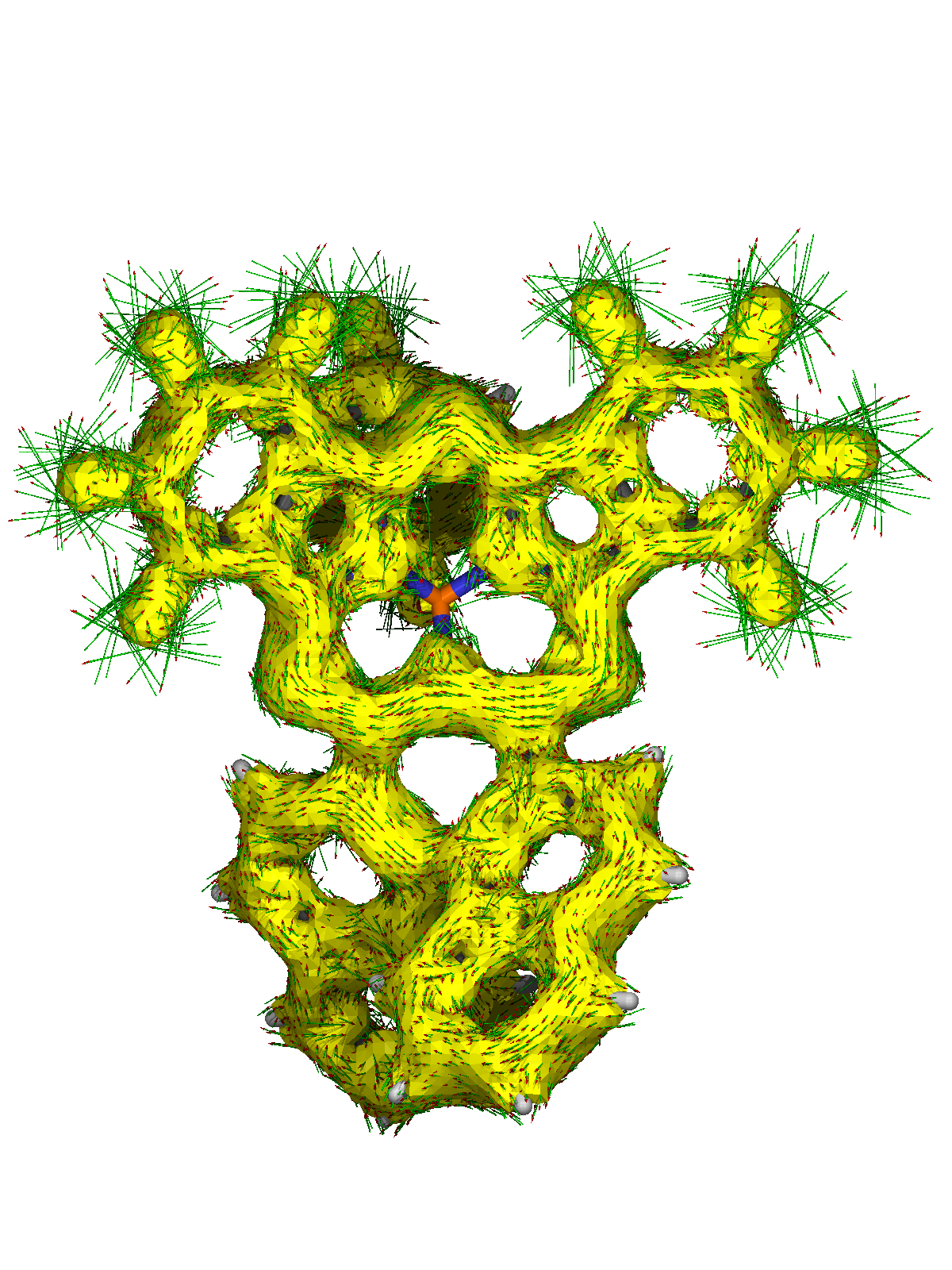


**Figure S6.** AICD plot at isovalue of 0.05 of (*S*)-**1** (bottom view).


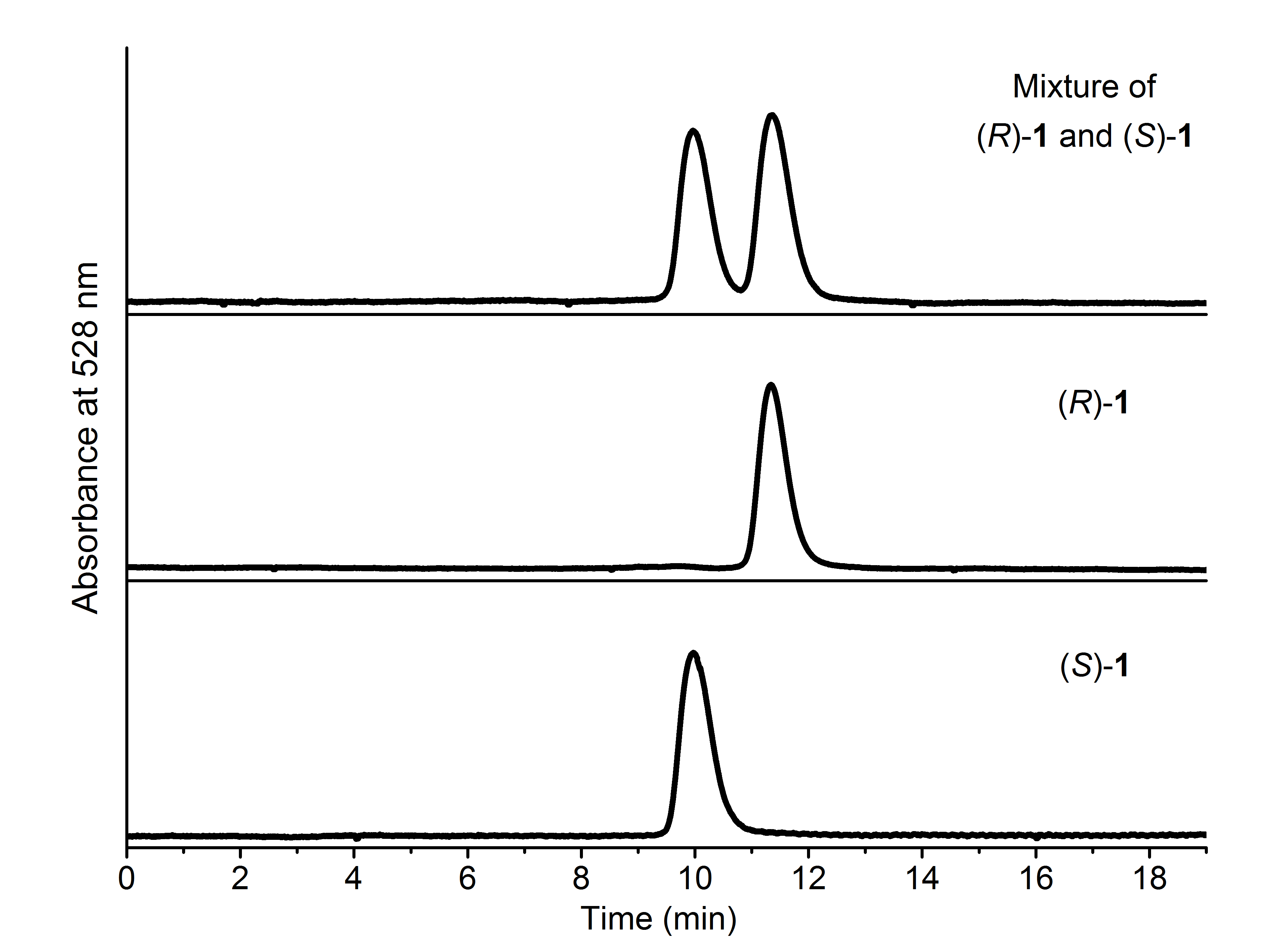


**Figure S7.** HPLC chromatogram of an arbitrary mixture of (*R*)-**1**/(*S*)-**1** (top), (*R*)-**1** (middle), and (*S*)-**1** (bottom) using a preparative CHIRALPAK IA-3 column.


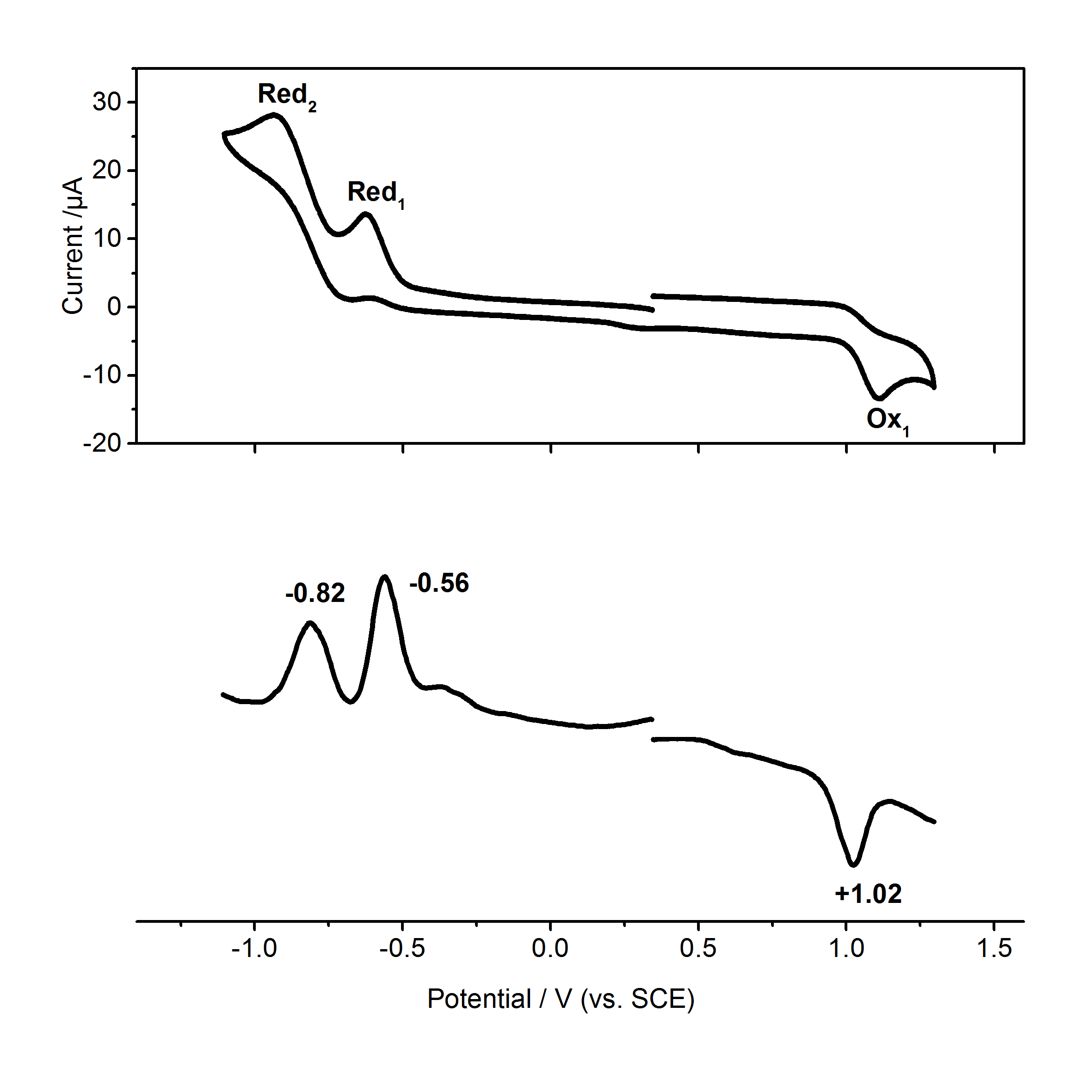


**Figure S8.** Cyclic voltammogram and differential pulse voltammogram of **1** in CH_2_Cl_2_ containing 0.1 M [NBu_4_][ClO_4_] at scan rate of 75 mV⋅s^−1^.


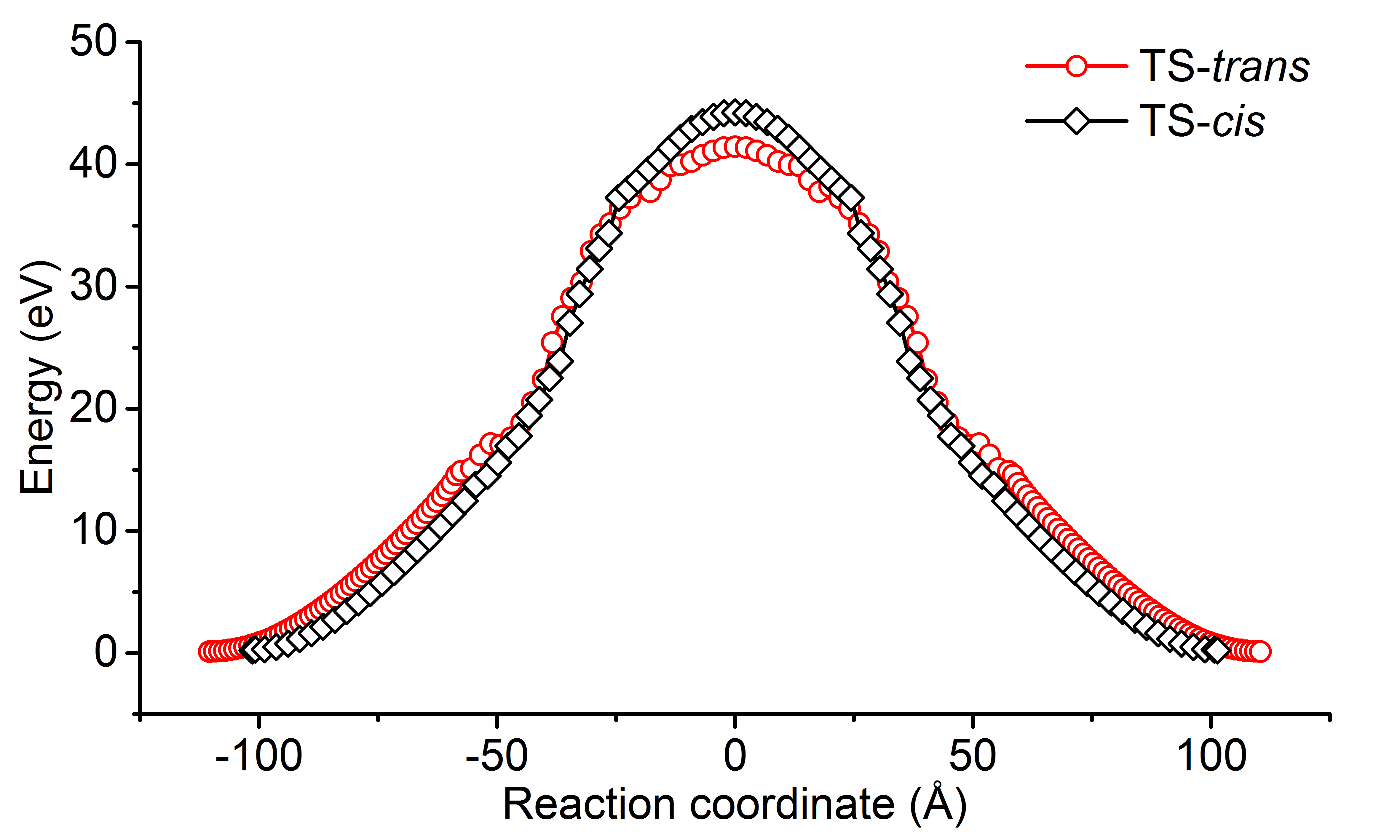


**Figure S9.** IRC paths of transition structures mentioned in Figure 5.

**Figures of ^1^H and ^13^C NMR spectra of compounds 3-7**

(*R*/*S*)-1,1'-binaphthalene-2,2'-diyl bis(trifluoromethanesulfonate) (**4**)


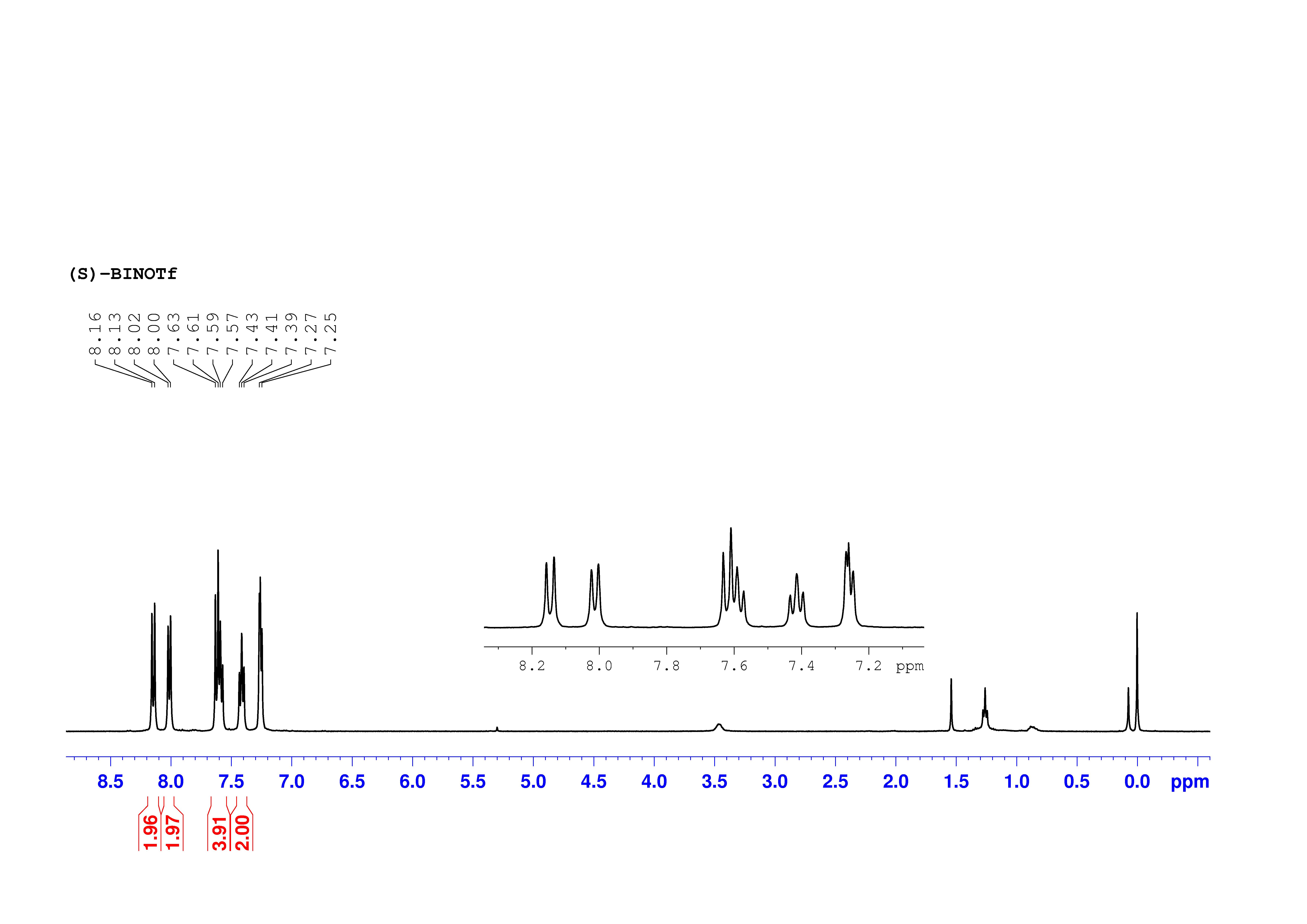


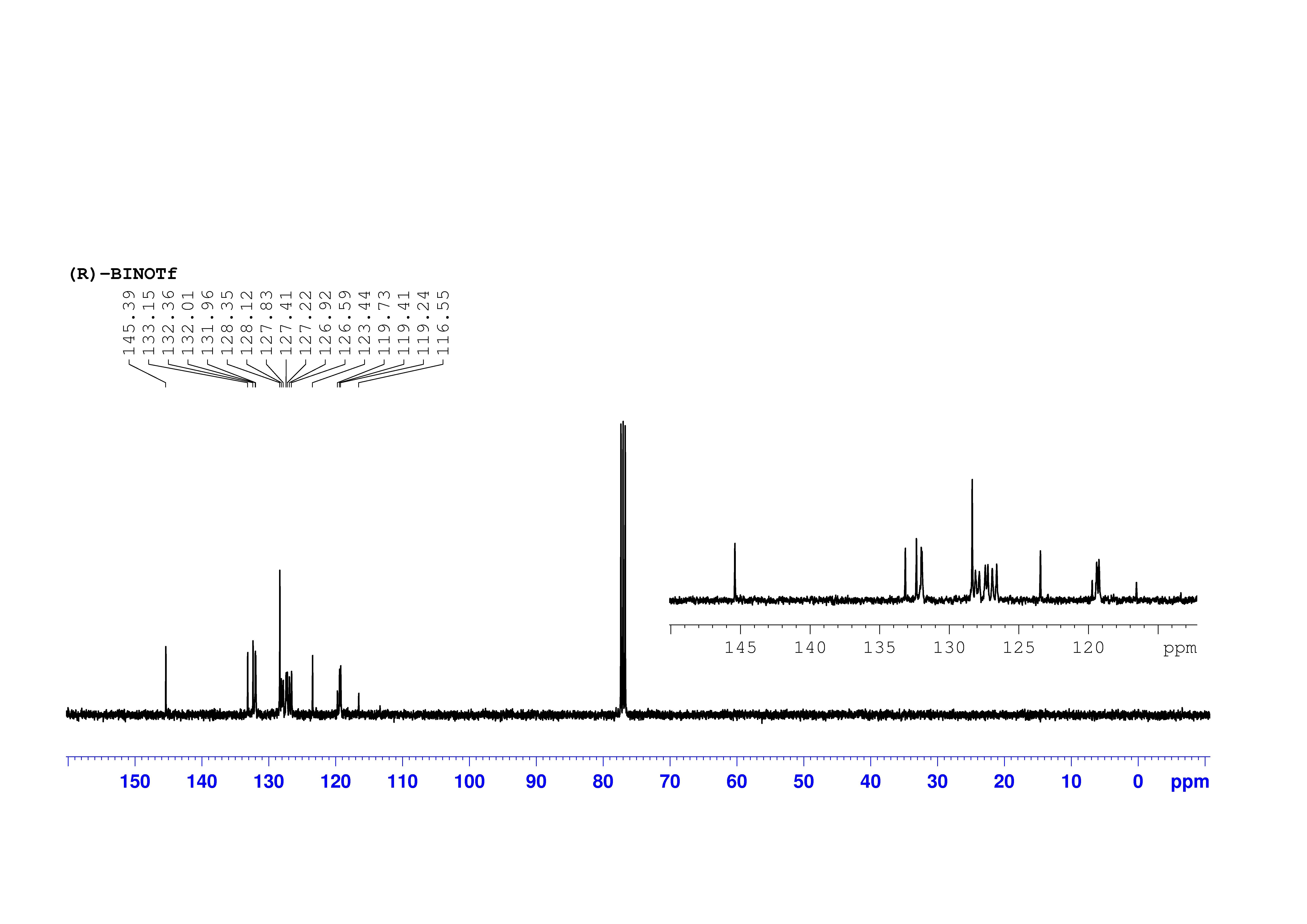


(*R*/*S*)-2,2'-dimethyl-1,1'-binaphthalene (**5**)


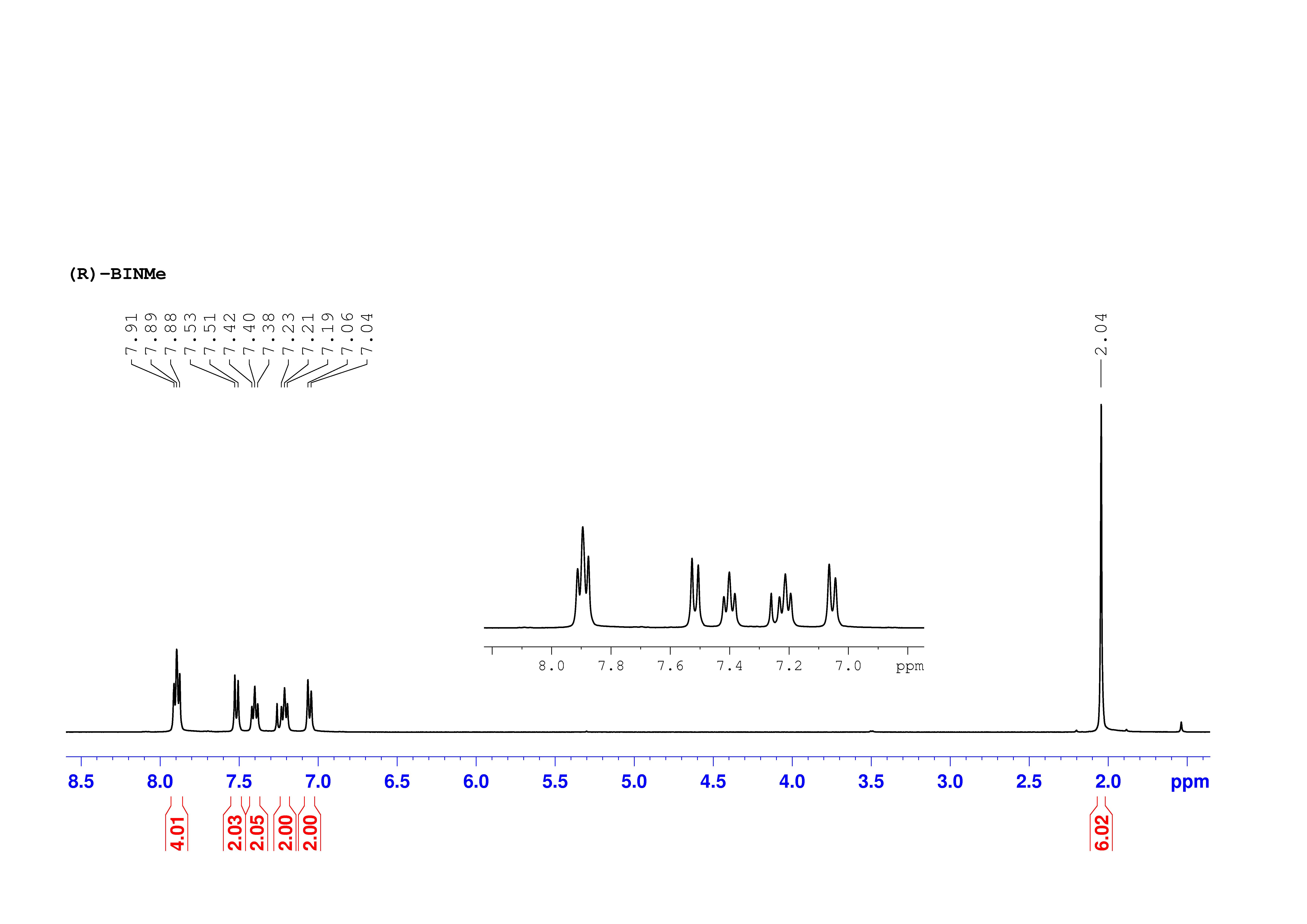


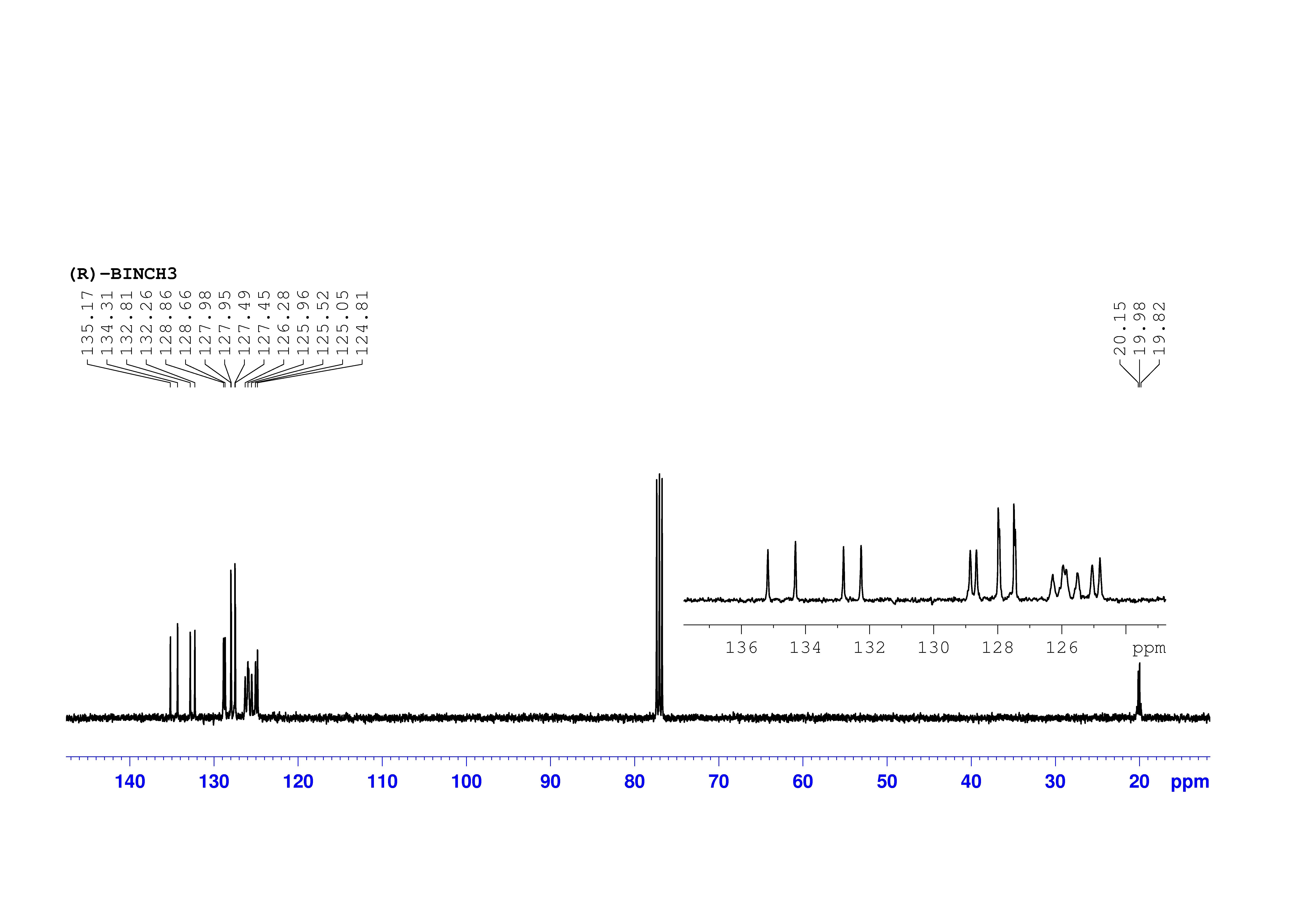


(*R*/*S*)-2,2'-bis(dibromomethyl)-1,1'-binaphthalene (**6**)


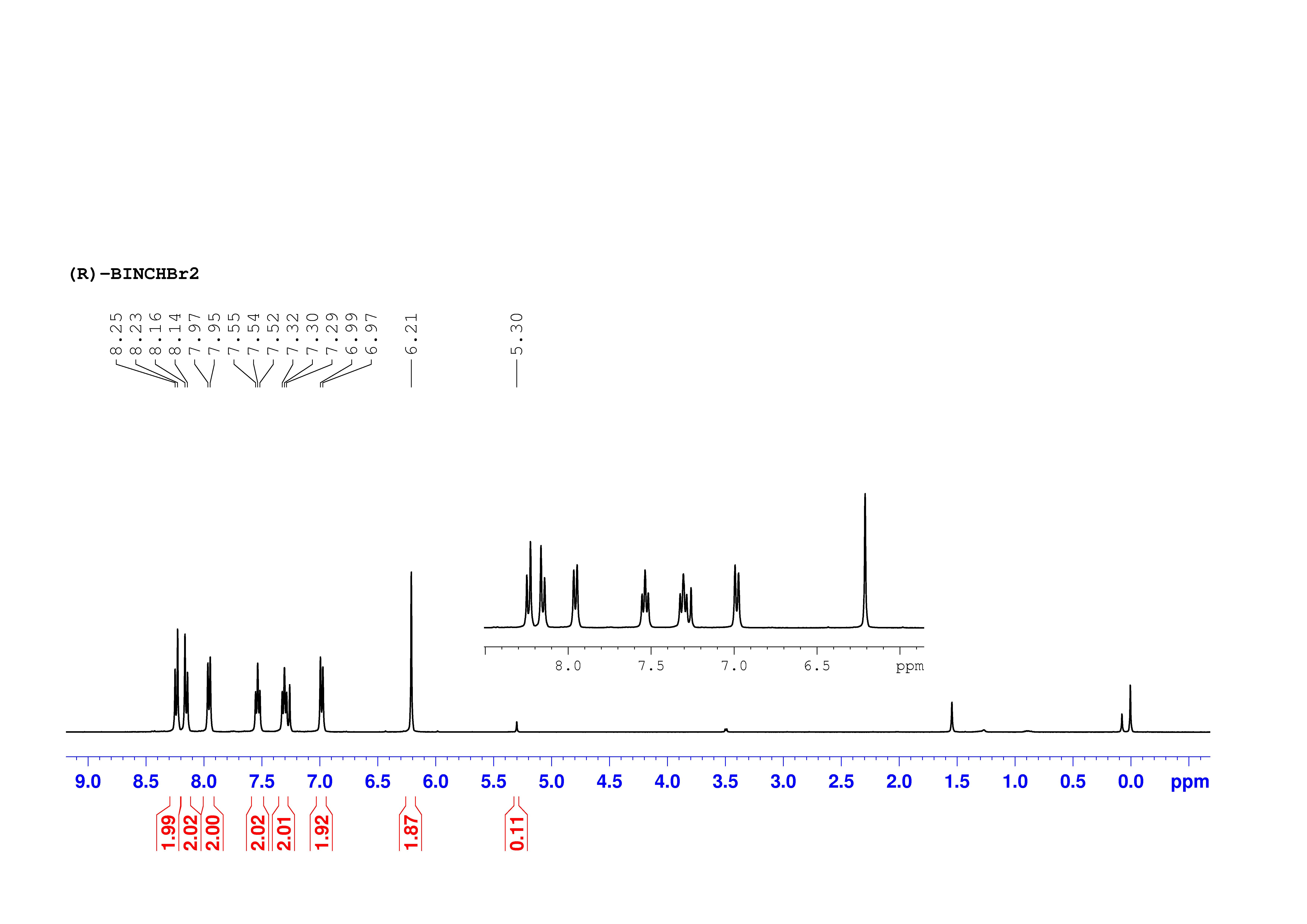


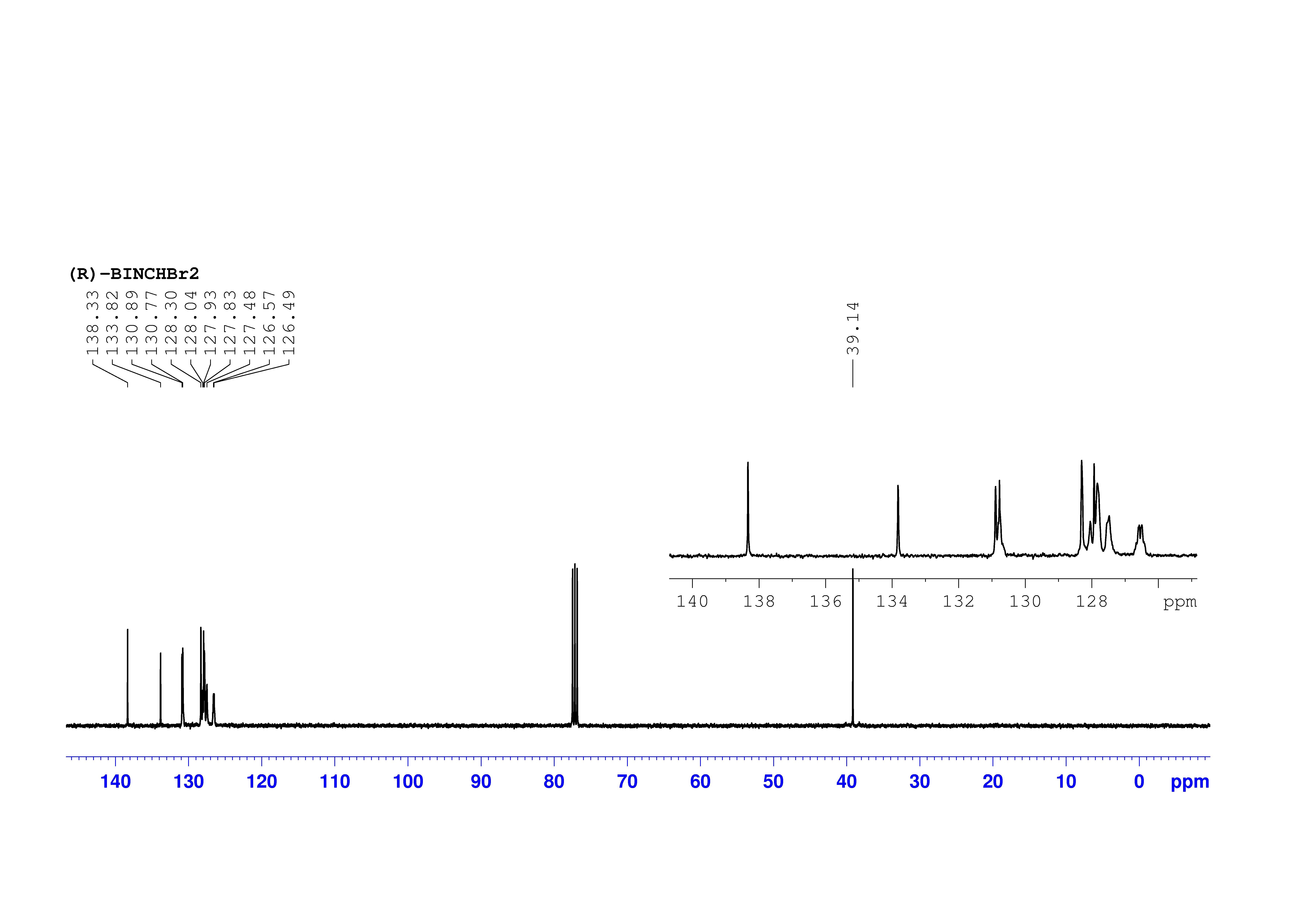


(R/S)-1,1'-binaphthalene-2,2'-dicarboxylic acid (**7**)


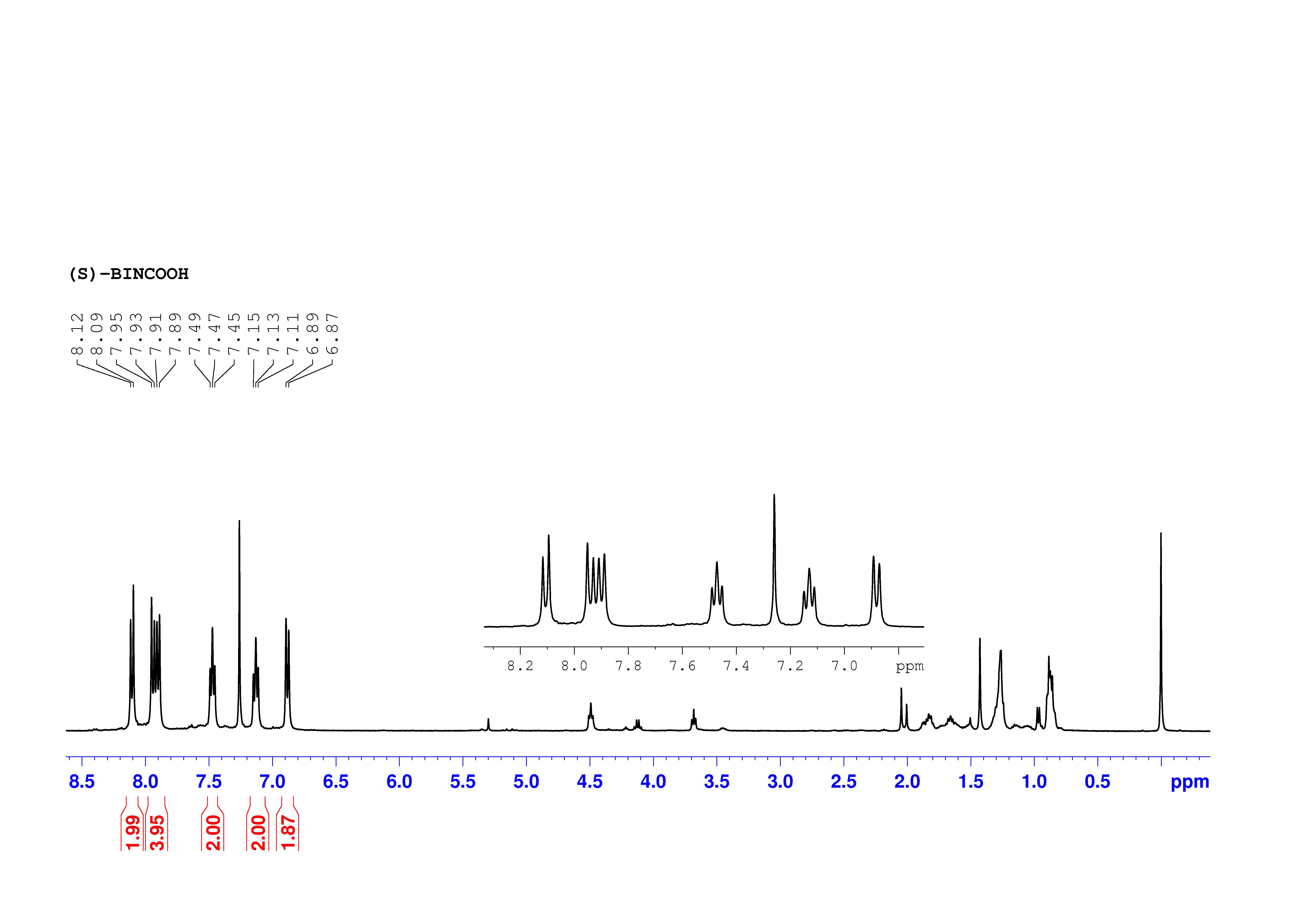

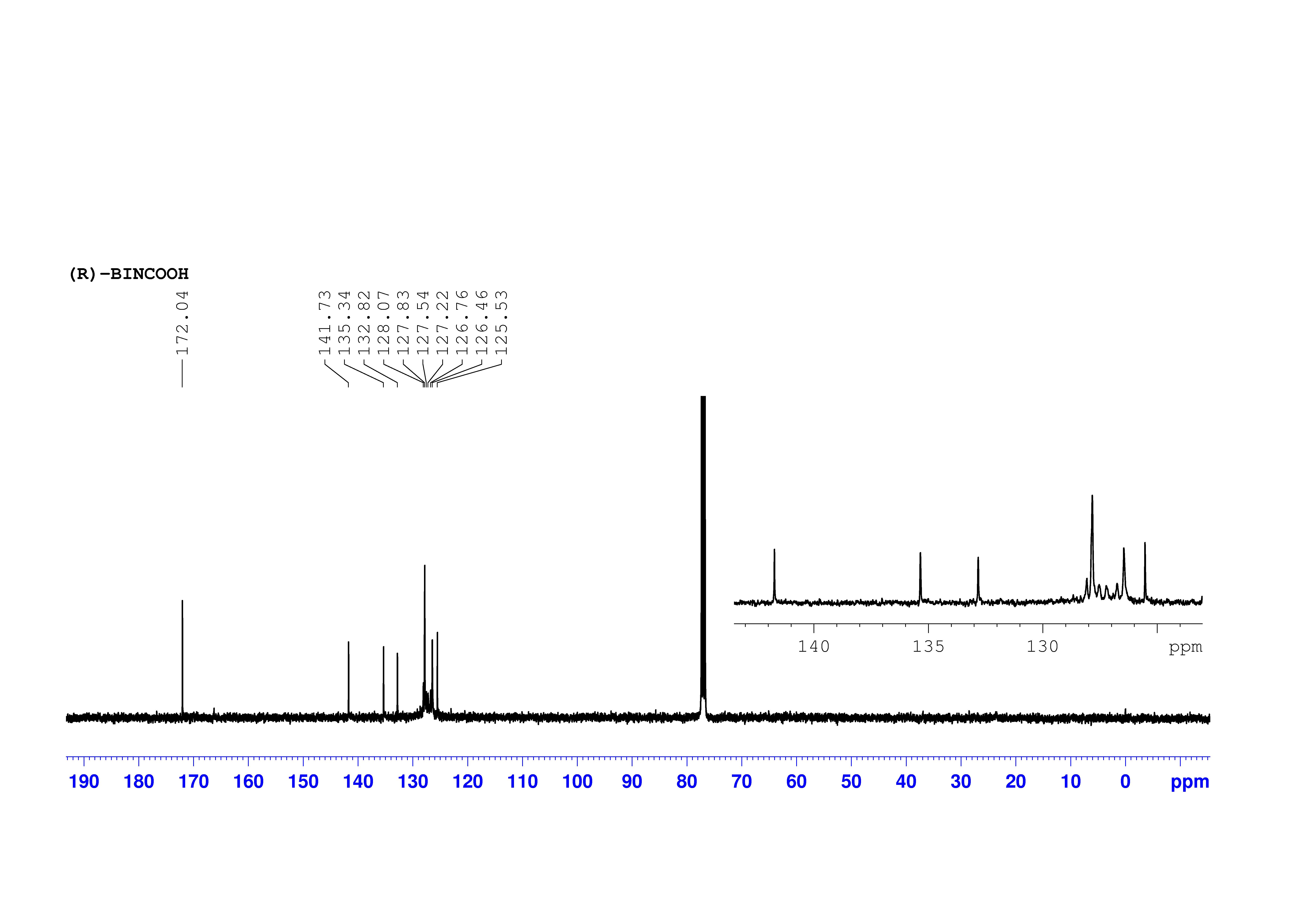


(*R*/*S*)-1,1'-binaphthalene-2,2'-dicarbonitrile (**3**)


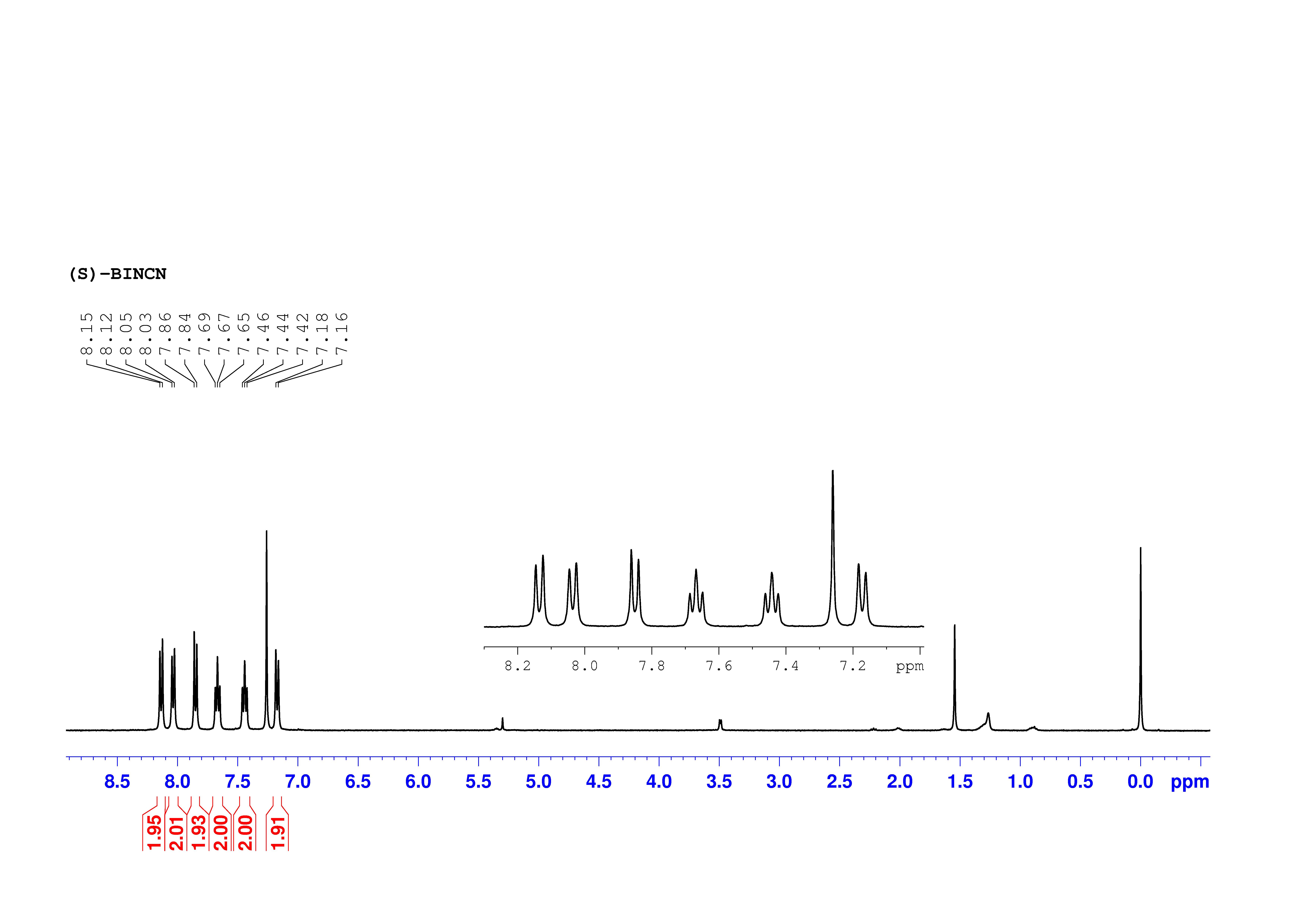

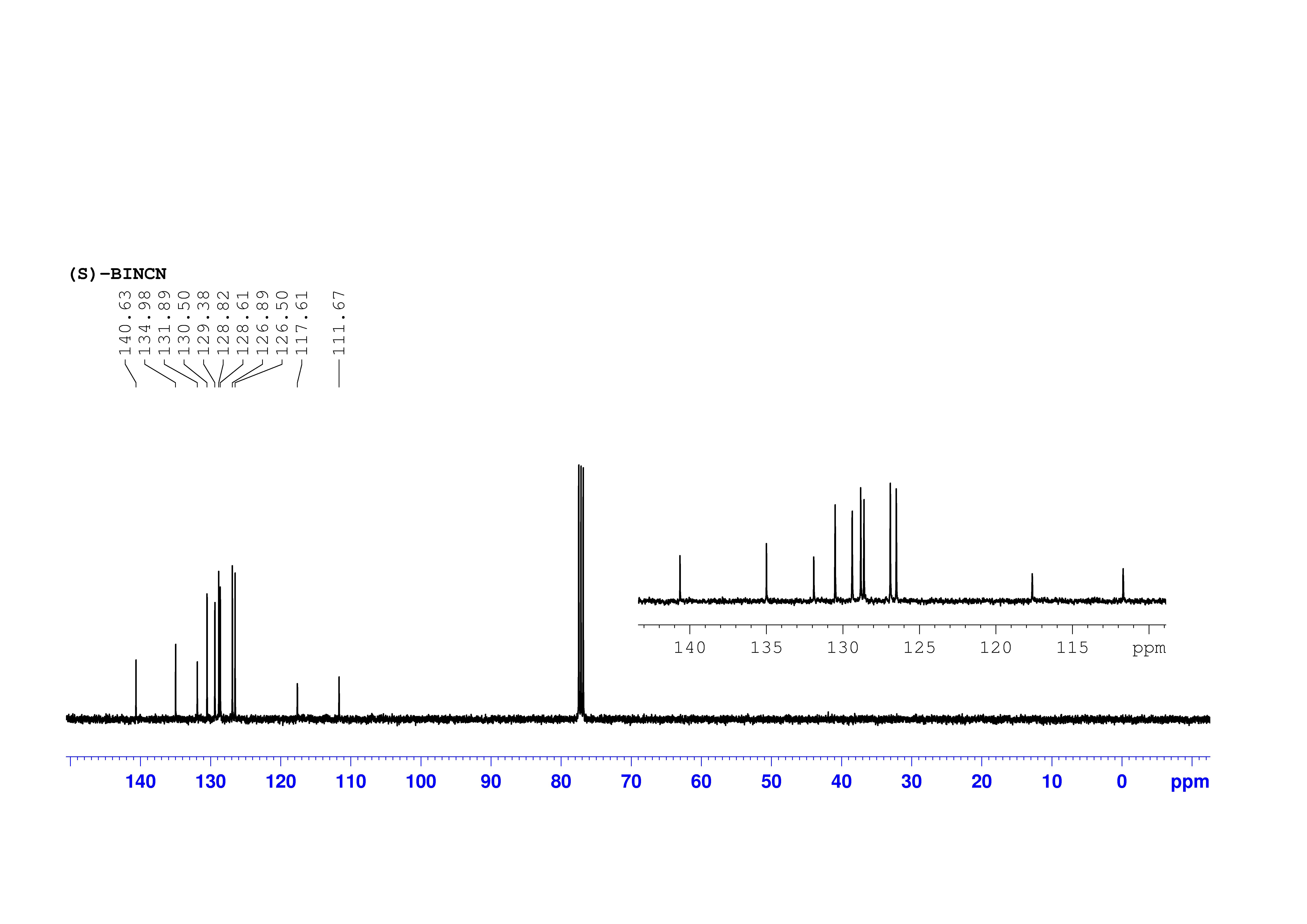


**Table S1.** ^1^H NMR data (*δ*) for **1** recorded in CDCl_3_ at 25°C.

|  | H*_a_* | H*_b_* | H*_c_* | H*_d_* | H*_e_* | H*_f_* | H*_g_* |
| --- | --- | --- | --- | --- | --- | --- | --- |
| **Exp.** | 7.76 | 8.16 | 8.00 | 7.62 | 7.31 | 7.11 | 7.02 |
|  | H*_h_* | H*_i_* | H*_j_* | H*_k_* | H*_l_* | H*_p_*, H*_p_*' | H*_q_*, H*_q_*' |
| **Exp.** | 7.20 | 7.55 | 7.92 | 7.85 | 8.16 | 5.37 | 6.28 |

**Table S2.** **Calculation of ratio of CD intensity against coefficient factor *υ*_N_*υ*/(*υ*_N_^2^−*υ*^2^).**

| Electron transition | *λ* (nm) | *υ* (10^15^ s^−1^) |  | *θ* (deg) |  | Relative ratio[*^b^*] |
| --- | --- | --- | --- | --- | --- | --- |
| Q_1_ | 698 | 0.43 | 0.6 | 838 | 1.4 | 1.0 |
| Q_2_ | 530 | 0.56 | 0.9 | 1054 | 1.1 | 0.8 |
| h-Soret | 348 | 0.86 | 6.4 | 877 | 0.1 | 0.07 |

[*^a^*] The ratio of CD intensity against the factor *υ*_N_*υ*/(*υ*_N_^2^−*υ*^2^), where the unit is omitted.

[*^b^*] Relative ratio is obtained with normalization of Q_1_ transition.

**Table S3.** Electric transition dipole moment ***μ*** and magnetic transition dipole moment ***m*** decomposition into **Mac** and **Bin** group contributions. All the listed values are in atomic unit.

|  | ***μ****_x_* | ***μ****_y_* | ***μ****_z_* | ***m****_x_* | ***m****_y_* | ***m****_z_* |
| --- | --- | --- | --- | --- | --- | --- |
| Q_1_-**1** | −0.061 | −1.840 | 0.188 | 0.125 | −0.001 | 1.965 |
| **Mac** | 0.047 | −1.955 | 0.384 | 0.132 | 0.246 | 1.116 |
| **Bin** | −0.106 | 0.111 | −0.197 | −0.008 | −0.246 | 0.848 |
| Q_2_-**1** | −2.644 | −0.030 | 0.306 | −0.159 | −0.019 | −0.285 |
| **Mac** | −1.190 | 0.002 | 0.303 | −0.037 | −0.049 | −0.191 |
| **Bin** | −1.453 | −0.032 | 0.004 | −0.122 | 0.031 | −0.093 |
| l-Soret-**1** | 0.033 | 1.504 | −0.258 | −0.099 | −0.328 | 0.751 |
| **Mac** | −0.007 | 1.365 | −0.338 | −0.111 | −0.006 | −0.208 |
| **Bin** | 0.041 | 0.138 | 0.081 | 0.012 | −0.323 | 0.956 |
| h-Soret-**1** | −1.178 | −0.162 | 0.013 | −0.310 | −0.049 | −0.327 |
| **Mac** | −0.300 | −0.101 | 0.133 | −0.180 | −0.085 | −0.375 |
| **Bin** | −0.875 | −0.060 | −0.120 | −0.126 | 0.035 | 0.052 |

**Z-matrices of optimized structures**

(1) (*S*)-**1**

B

C 1 2.54730794

C 2 1.44713519 1 133.2424816

C 3 1.38601162 2 132.5455106 1 −177.4916574 0

C 4 1.383266 3 118.7883294 2 −175.0706598 0

C 5 1.39864589 4 121.2423637 3 1.0502782 0

C 6 1.38131516 5 120.6620815 4 −0.2822284 0

C 7 1.38541394 6 118.7528168 5 −1.1072579 0

C 8 1.44684213 7 132.2472984 6 175.2176617 0

C 9 2.3203388 8 155.5297718 7 −42.8949284 0

C 1 2.42182108 9 116.3027352 8 147.6978331 0

C 11 2.31485318 1 62.6007714 9 −129.8630585 0

C 12 1.43617563 11 159.449111 1 158.8285959 0

C 13 1.38938669 12 132.691896 11 29.6998393 0

C 14 1.37789262 13 118.872508 12 −177.282691 0

C 15 1.40237976 14 120.8347876 13 0.5885237 0

C 16 1.3793939 15 121.1683971 14 0.1623162 0

C 17 1.38937711 16 118.8980269 15 −0.8462451 0

C 18 1.43923915 17 132.6177099 16 176.086573 0

N 9 1.34052266 8 105.9864462 7 179.5045475 0

N 12 1.3383336 11 91.860724 1 7.4909945 0

N 10 1.36141696 9 93.5604642 8 −140.878958 0

N 2 1.3198335 1 94.8407528 20 167.9040998 0

N 11 1.31739379 1 90.6088041 20 −116.0815106 0

N 9 1.32256288 8 131.0506757 7 −13.5153922 0

C 11 1.46950584 1 136.095786 20 109.7865021 0

C 26 1.38065139 11 122.9015608 1 −79.8862782 0

C 26 1.41586468 11 116.0129275 1 91.7211687 0

C 27 1.4297494 26 119.0519291 11 161.8262389 0

C 28 1.36125574 26 120.616161 11 −167.642597 0

H 28 1.08587846 26 118.0129818 11 11.6488653 0

C 29 1.41708521 27 122.6711717 26 −170.128966 0

C 30 1.41234942 28 120.5952688 26 2.9041662 0

H 30 1.08864153 28 120.4239808 26 −179.8396412 0

C 32 1.36719145 29 121.1114632 27 178.9762028 0

H 32 1.08692951 29 119.3512125 27 0.2649095 0

C 33 1.41285519 30 121.3107089 28 174.8384171 0

C 37 1.3665006 33 120.919579 30 −177.7513726 0

H 35 1.08734324 32 119.7222488 29 −178.9911501 0

H 37 1.08889191 33 118.3787754 30 1.3421713 0

H 38 1.08729923 37 120.2988715 33 179.5161889 0

C 10 1.4739681 9 140.9808964 8 12.9231021 0

C 42 1.38677103 10 124.2315033 9 −177.7258069 0

C 42 1.42106182 10 115.7824399 9 −4.079895 0

C 43 1.43309093 42 118.9973912 10 163.0918882 0

C 44 1.35885624 42 121.2825914 10 −169.2680658 0

H 44 1.08501898 42 117.7131243 10 10.6837175 0

C 45 1.41785212 43 122.4608993 42 −169.1237393 0

C 46 1.41229859 44 120.5748011 42 2.9625305 0

H 46 1.08861367 44 120.3306212 42 −179.6574567 0

C 48 1.36751656 45 121.1564097 43 179.1391098 0

H 48 1.08655368 45 119.4898801 43 0.4953251 0

C 49 1.41203523 46 121.3464337 44 174.6074249 0

C 53 1.36685667 49 120.8343764 46 −177.5311981 0

H 51 1.08729958 48 119.6759622 45 −178.9913288 0

H 53 1.08883363 49 118.4011207 46 1.5307633 0

H 54 1.08736936 53 120.2912407 49 179.5047865 0

O 1 1.42516577 20 116.4655063 9 90.8465309 0

C 58 1.36413347 1 117.9252396 20 60.6319612 0

C 59 1.39071448 58 120.1299399 1 −91.1620707 0

C 59 1.39119489 58 120.0936021 1 88.8988717 0

C 60 1.38615592 59 120.2508301 58 −179.9523882 0

H 60 1.08830147 59 118.8659697 58 −0.4422772 0

C 61 1.3860465 59 120.2679553 58 179.9771857 0

H 61 1.08868286 59 118.868273 58 0.5166597 0

C 62 1.38159359 60 118.9988222 59 −0.1188580 0

H 62 1.08659656 60 121.6340726 59 179.8257738 0

H 64 1.08661307 61 121.6563357 59 −179.8740174 0

F 66 1.33759683 62 119.1456739 60 −179.9807739 0

F 17 1.32010334 16 119.5096517 15 179.5207608 0

F 16 1.32149673 15 118.5234755 14 −179.8356065 0

F 15 1.32217381 14 120.479926 13 −179.2687037 0

F 14 1.32168805 13 121.3960352 12 2.964011 0

F 7 1.32164703 6 119.5643447 5 179.1070527 0

F 6 1.32122893 5 118.8880461 4 179.6475222 0

F 5 1.31985879 4 120.155879 3 −179.1626744 0

F 4 1.31888026 3 121.9096028 2 5.0671302 0

(2) (*R*)-**1**

B

C 1 2.54730818

C 2 1.44713562 1 133.2424687

C 3 1.38601208 2 132.5455037 1 177.4916803 0

C 4 1.38326545 3 118.7883011 2 175.0707143 0

C 5 1.39864608 4 121.2423961 3 −1.05028697 0

C 6 1.3813153 5 120.6620704 4 0.28223613 0

C 7 1.38541345 6 118.7528117 5 1.10728168 0

C 8 1.44684201 7 132.247279 6 −175.2176436 0

C 9 2.3203388 8 155.5298068 7 42.89487347 0

C 1 2.42182082 9 116.3027348 8 −147.6978559 0

C 11 2.31485349 1 62.6007436 9 129.863076 0

C 12 1.43617587 11 159.4490808 1 −158.8286034 0

C 13 1.38938651 12 132.6918819 11 −29.69980794 0

C 14 1.37789351 13 118.8725023 12 177.2826753 0

C 15 1.40237943 14 120.8347306 13 −0.58853372 0

C 16 1.37939346 15 121.168476 14 −0.16233248 0

C 17 1.38937719 16 118.8980036 15 0.84625025 0

C 18 1.43923897 17 132.6177517 16 −176.0865788 0

N 9 1.34052354 8 105.9864566 7 −179.5045796 0

N 12 1.3383332 11 91.86073182 1 −7.4910096 0

N 10 1.36141744 9 93.56048192 8 140.8789709 0

N 2 1.31983352 1 94.84075112 20 −167.9040446 0

N 11 1.3173941 1 90.60879671 20 116.0815332 0

N 9 1.32256281 8 131.0507296 7 13.51531363 0

C 11 1.46950552 1 136.0957703 20 −109.7865377 0

C 26 1.38065126 11 122.9015632 1 79.88632434 0

C 26 1.41586461 11 116.0129621 1 −91.72115375 0

C 27 1.42974926 26 119.0519499 11 −161.8262682 0

C 28 1.36125586 26 120.6162002 11 167.6426482 0

H 28 1.0858791 26 118.0129924 11 −11.64883998 0

C 29 1.41708513 27 122.6711679 26 170.128924 0

C 30 1.41234986 28 120.595227 26 −2.90421453 0

H 30 1.08864127 28 120.4239771 26 179.8397191 0

C 32 1.36719177 29 121.1114721 27 −178.976236 0

H 32 1.08692984 29 119.351229 27 −0.26484121 0

C 33 1.41285564 30 121.3106725 28 −174.838366 0

C 37 1.36650055 33 120.9195411 30 177.7514246 0

H 35 1.08734264 32 119.7222713 29 178.9911584 0

H 37 1.08889218 33 118.3787752 30 −1.34216584 0

H 38 1.08729935 37 120.298849 33 −179.5161715 0

C 10 1.4739688 9 140.9808703 8 −12.92308176 0

C 42 1.38677024 10 124.2314769 9 177.7257878 0

C 42 1.42106152 10 115.782417 9 4.07988588 0

C 43 1.43309038 42 118.9974064 10 −163.0919158 0

C 44 1.35885599 42 121.2825621 10 169.268076 0

H 44 1.0850184 42 117.7131626 10 −10.68372411 0

C 45 1.41785253 43 122.4609315 42 169.1237553 0

C 46 1.41229936 44 120.5748151 42 −2.96255335 0

H 46 1.08861355 44 120.3306749 42 179.6574642 0

C 48 1.36751643 45 121.1564038 43 −179.1390567 0

H 48 1.08655344 45 119.4898646 43 −0.49531153 0

C 49 1.41203433 46 121.3464606 44 −174.6074309 0

C 53 1.36685679 49 120.8344281 46 177.5312147 0

H 51 1.08730036 48 119.6759371 45 178.9913561 0

H 53 1.08883415 49 118.4011457 46 −1.5307349 0

H 54 1.0873686 53 120.291269 49 −179.50476 0

O 1 1.42516631 20 116.4654621 9 −90.84657063 0

C 58 1.3641327 1 117.9252628 20 −60.63195433 0

C 59 1.39071451 58 120.129965 1 91.16208785 0

C 59 1.3911953 58 120.0936177 1 −88.89887917 0

C 60 1.38615556 59 120.2508936 58 179.95236 0

H 60 1.08830191 59 118.8659274 58 0.44230325 0

C 61 1.38604602 59 120.2679492 58 −179.9772089 0

H 61 1.08868256 59 118.8682977 58 −0.51659943 0

C 62 1.38159401 60 118.9987907 59 0.11887829 0

H 62 1.08659691 60 121.6341169 59 −179.8257629 0

H 64 1.08661322 61 121.6563237 59 179.8740436 0

F 66 1.33759706 62 119.1456808 60 179.9807294 0

F 17 1.3201027 16 119.5097007 15 −179.5207504 0

F 16 1.32149773 15 118.5234271 14 179.8355899 0

F 15 1.32217377 14 120.4799204 13 179.2687043 0

F 14 1.32168798 13 121.3960725 12 −2.9640536 0

F 7 1.32164671 6 119.5643007 5 −179.1070695 0

F 6 1.32122917 5 118.8880415 4 −179.6475251 0

F 5 1.31985891 4 120.1558824 3 179.1626749 0

F 4 1.31888044 3 121.9095721 2 −5.06706041 0

(3) TS-*cis* (in Figure 5)

B

C 1 2.52635439

C 2 1.44111614 1 133.7303232

C 3 1.39268027 2 132.1593301 1 179.8240712 0

H 4 1.08739081 3 120.2620742 2 4.22565041 0

C 4 1.38089081 3 118.2717478 2 −175.983305 0

C 6 1.40401963 4 121.4415356 3 0.96207988 0

C 7 1.38050021 6 121.0456881 4 0.11599505 0

H 8 1.08766819 7 121.4505989 6 179.328306 0

C 8 1.39261668 7 118.2271802 6 −1.10810623 0

C 10 1.43954046 8 132.2975148 7 176.8488034 0

C 11 2.32244571 10 157.2053226 8 −36.19271373 0

C 1 2.46249873 11 112.0028742 10 140.5954929 0

C 13 2.32244571 1 61.59599412 11 −130.727546 0

C 14 1.43954046 13 157.2053226 1 152.9169042 0

C 15 1.39261668 14 132.2975148 13 36.19271373 0

H 16 1.08766819 15 120.3207737 14 3.58249134 0

C 16 1.38050021 15 118.2271802 14 −176.8488034 0

C 18 1.40401963 16 121.0456881 15 1.10810623 0

C 19 1.38089081 18 121.4415356 16 −0.11599505 0

H 20 1.08739081 19 121.4658382 18 179.2495159 0

C 20 1.39268027 19 118.2717478 18 −0.96207988 0

C 22 1.44111614 20 132.1593301 19 175.983305 0

N 11 1.33996147 10 106.5112389 8 179.2251019 0

N 14 1.33996147 13 92.33307447 1 6.70327199 0

N 12 1.37088206 11 93.03692865 10 −138.8868921 0

N 2 1.32603502 1 94.04253281 24 169.2539957 0

N 13 1.3257835 1 89.85015988 24 −116.0202862 0

N 12 1.3257835 11 29.25777624 10 43.49475732 0

H 18 1.08786633 16 119.6860595 15 −179.2733344 0

H 19 1.08753153 18 119.0174176 16 179.5887518 0

H 7 1.08786633 6 119.2671691 4 179.7361275 0

H 6 1.08753153 4 119.5403944 3 −179.3346882 0

Cl 1 1.87631019 24 113.2760165 11 94.02859087 0

C 13 1.47174646 1 151.7276824 24 92.35505292 0

C 35 1.41056319 13 124.5044478 1 2.86780765 0

C 35 1.41278784 13 114.4583167 1 176.3685781 0

C 36 1.45578369 35 113.1978982 13 148.7649646 0

C 37 1.3596163 35 121.6192635 13 −170.937853 0

H 37 1.08420652 35 117.2973233 13 11.45984952 0

C 38 1.41123685 36 124.8026483 35 −143.880954 0

C 39 1.40866733 37 118.3884155 35 15.10705048 0

H 39 1.08796045 37 121.3889582 35 −172.4940023 0

C 41 1.37147982 38 122.4897307 36 −177.1328819 0

H 41 1.07889438 38 118.9564328 36 11.07358362 0

C 42 1.41251014 39 120.21068 37 164.3166798 0

C 46 1.36666983 42 121.1764627 39 −166.2257216 0

H 44 1.08745237 41 119.3911413 38 −177.4351248 0

H 46 1.08888668 42 117.9455394 39 9.78937325 0

H 47 1.08711869 46 120.6967941 42 178.2680689 0

C 12 1.47174646 11 144.0327389 10 43.43994225 0

C 51 1.41056319 12 124.5044478 11 145.5998201 0

C 51 1.41278784 12 114.4583167 11 −27.90095039 0

C 52 1.45578369 51 113.1978982 12 −148.7649646 0

C 53 1.3596163 51 121.6192635 12 170.937853 0

H 53 1.08420652 51 117.2973233 12 −11.45984952 0

C 54 1.41123685 52 124.8026483 51 143.880954 0

C 55 1.40866733 53 118.3884155 51 −15.10705048 0

H 55 1.08796045 53 121.3889582 51 172.4940023 0

C 57 1.37147982 54 122.4897307 52 177.1328819 0

H 57 1.07889438 54 118.9564328 52 −11.07358362 0

C 58 1.41251014 55 120.21068 53 −164.3166798 0

C 62 1.36666983 58 121.1764627 55 166.2257216 0

H 60 1.08745237 57 119.3911413 54 177.4351248 0

H 62 1.08888668 58 117.9455394 55 −9.78937325 0

H 63 1.08711869 62 120.6967941 58 −178.2680689 0

(4) TS-*trans* (in Figure 5)

B

C 1 2.52423598

C 2 1.44231696 1 133.3381387

C 3 1.3920703 2 132.0925774 1 179.8507523 0

H 4 1.08732657 3 120.2950189 2 3.83373131 0

C 4 1.38100283 3 118.2579189 2 −176.2913461 0

C 6 1.40404916 4 121.4379191 3 1.0460532 0

C 7 1.380468 6 121.0868406 4 0.04047209 0

H 8 1.08756595 7 121.5560341 6 178.9916657 0

C 8 1.39217058 7 118.1932699 6 −1.18618176 0

C 10 1.44049981 8 132.3022669 7 176.7937276 0

C 11 2.32266295 10 156.7280847 8 −39.61561226 0

C 1 2.4459416 11 113.7207394 10 140.57449 0

C 13 2.32266295 1 61.52027071 11 −134.4658977 0

C 14 1.44049981 13 156.7280847 1 150.5985491 0

C 15 1.39217058 14 132.3022669 13 39.61561226 0

H 16 1.08756595 15 120.250456 14 3.38171045 0

C 16 1.380468 15 118.1932699 14 −176.7937276 0

C 18 1.40404916 16 121.0868406 15 1.18618176 0

C 19 1.38100283 18 121.4379191 16 −0.04047209 0

H 20 1.08732657 19 121.4469404 18 179.0805367 0

C 20 1.3920703 19 118.2579189 18 −1.0460532 0

C 22 1.44231696 20 132.0925774 19 176.2913461 0

N 11 1.34038375 10 106.3037129 8 179.5429599 0

N 14 1.34038375 13 92.07163678 1 7.93338985 0

N 12 1.36669557 11 93.01956112 10 −135.0431728 0

N 2 1.32696084 1 94.13706533 24 170.4687197 0

N 13 1.32772403 1 89.52080685 24 −119.5545709 0

N 12 1.32772403 11 29.31327346 10 48.18166629 0

H 18 1.08733039 16 119.7150893 15 −179.1401132 0

H 19 1.08772207 18 119.0600286 16 179.6221516 0

H 7 1.08733039 6 119.1972785 4 179.7158383 0

H 6 1.08772207 4 119.5012012 3 −179.292783 0

Cl 1 1.87727682 24 113.1746362 11 90.21228188 0

C 13 1.47496619 1 154.5207548 24 83.60173833 0

C 35 1.40724861 13 126.456506 1 −71.14559482 0

C 35 1.4151956 13 112.3508995 1 112.7924704 0

C 36 1.46087009 35 112.8838077 13 −152.2991932 0

C 37 1.35771264 35 121.572645 13 174.8369144 0

H 37 1.0849725 35 117.4914768 13 −8.81764252 0

C 38 1.41043266 36 125.0120727 35 143.17722 0

C 39 1.40916288 37 118.2585324 35 −15.8175976 0

H 39 1.08783466 37 121.4324487 35 172.0077141 0

C 41 1.37246177 38 122.6389013 36 176.9966235 0

H 41 1.07869115 38 119.0155307 36 −11.0733718 0

C 42 1.411842 39 119.9179371 37 −164.0040801 0

C 46 1.36695154 42 121.1838551 39 165.2695196 0

H 44 1.08748198 41 119.330528 38 177.7337708 0

H 46 1.08884839 42 117.9373615 39 −10.66759641 0

H 47 1.08717367 46 120.7555984 42 −178.3544219 0

C 12 1.47496619 11 142.8658209 10 39.38980839 0

C 51 1.40724861 12 126.456506 11 −129.6112444 0

C 51 1.4151956 12 112.3508995 11 46.45069036 0

C 52 1.46087009 51 112.8838077 12 152.2991932 0

C 53 1.35771264 51 121.572645 12 −174.8369144 0

H 53 1.0849725 51 117.4914768 12 8.81764252 0

C 54 1.41043266 52 125.0120727 51 −143.17722 0

C 55 1.40916288 53 118.2585324 51 15.8175976 0

H 55 1.08783466 53 121.4324487 51 −172.0077141 0

C 57 1.37246177 54 122.6389013 52 −176.9966235 0

H 57 1.07869115 54 119.0155307 52 11.0733718 0

C 58 1.411842 55 119.9179371 53 164.0040801 0

C 62 1.36695154 58 121.1838551 55 −165.2695196 0

H 60 1.08748198 57 119.330528 54 −177.7337708 0

H 62 1.08884839 58 117.9373615 55 10.66759641 0

H 63 1.08717367 62 120.7555984 58 178.3544219 0

**References for supporting information**

[1] P. C. B. Page, B. R. Buckley, M. M. Farah, A. J. Blacker, *Eur. J. Org. Chem.* **2009**, *2009*, 3413-3426.

[2] Y. Xia, Z. Liu, Q. Xiao, P. Qu, R. Ge, Y. Zhang, J. Wang, *Angew. Chem. Int. Ed.* **2012**, *51*, 5714-5717.

[3] M. Schlosser, F. Bailly, *J. Am. Chem. Soc.* **2006**, *128*, 16042-16043.

[4] T. Hoshi, E. Nozawa, M. Katano, T. Suzuki, H. Hagiwara, *Tetrahedron Lett.* **2004**, *45*, 3485-3487.
